# A neurodegeneration gene, *WDR45*, links impaired ferritinophagy to iron accumulation

**DOI:** 10.1111/jnc.15548

**Published:** 2021-12-08

**Authors:** Luisa Aring, Eun‐Kyung Choi, Huira Kopera, Thomas Lanigan, Shigeki Iwase, Daniel J. Klionsky, Young Ah Seo

**Affiliations:** ^1^ Department of Nutritional Sciences University of Michigan School of Public Health Ann Arbor Michigan USA; ^2^ Department of Human Genetics University of Michigan Ann Arbor Michigan USA; ^3^ Vector Core Biomedical Research Core Facilities University of Michigan Ann Arbor Michigan USA; ^4^ Division of Rheumatology Department of Internal Medicine University of Michigan Ann Arbor Michigan USA; ^5^ Life Sciences Institute, and the Department of Molecular, Cellular and Developmental Biology University of Michigan Ann Arbor Michigan USA

**Keywords:** BPAN, ferritinophagy, iron, NBIA, neurodegeneration

## Abstract

Neurodegeneration with brain iron accumulation (NBIA) is a clinically and genetically heterogeneous group of neurodegenerative diseases characterized by the abnormal accumulation of brain iron and the progressive degeneration of the nervous system. One of the recently identified subtypes of NBIA is β‐propeller protein‐associated neurodegeneration (BPAN). BPAN is caused by *de novo* mutations in the *WDR45*/*WIPI4* (WD repeat domain 45) gene. WDR45 is one of the four mammalian homologs of yeast *Atg18*, a regulator of autophagy. WDR45 deficiency in BPAN patients and animal models may result in defects in autophagic flux. However, how WDR45 deficiency leads to brain iron overload remains unclear. To elucidate the role of WDR45, we generated a *WDR45*‐knockout (KO) SH‐SY5Y neuroblastoma cell line using CRISPR‐Cas9‐mediated genome editing. Using these cells, we demonstrated that the non‐TF (transferrin)‐bound iron pathway dominantly mediated the accumulation of iron. Moreover, the loss of WDR45 led to defects in ferritinophagy, a form of autophagy that degrades the iron storage protein ferritin. We showed that impaired ferritinophagy contributes to iron accumulation in *WDR45*‐KO cells. Iron accumulation was also detected in the mitochondria, which was accompanied by impaired mitochondrial respiration, elevated reactive oxygen species, and increased cell death. Thus, our study links WDR45 to specific iron acquisition pathways and ferritinophagy.

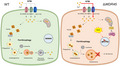

**Cover Image for this issue:** https://doi.org/10.1111/jnc.15388

## Graphical abstract

Schematic model for impact of WDR45 deficiency on iron accumulation, ferritinophagy, and cell death. WDR45‐deficient cells accumulate iron via the nontransferrin‐bound iron pathway (NTBI). The increased total iron levels were accompanied by an increase of the iron storage protein ferritin. WDR45 deficiency impairs ferritinophagy, the autophagic degradation of ferritin. Elevated iron levels were also detected in the mitochondria, which may impair mitochondrial function, elevate reactive oxygen species, and increase cell death.

**Abbreviations**
2‐DG: 2‐deoxy‐d‐glucose
8‐iso‐PGF: 8‐iso prostaglandin F2α
AChE: 8‐isoprostane‐acetylcholinesterase
ACTB: actin
APP: amyloid beta precursor protein
BPAN: beta‐propeller protein‐associated neurodegeneration
CASP3: caspase 3
CP: ceruloplasmin
CQ: chloroquine
CYCS: cytochrome *c*
DAPI: 4’,6‐diamidino‐2‐phenylindole
DCF: 2’,7'‐dichlorofluorescein
DFO: deferoxamine
ECAR: extracellular acidification rate
ELISA: enzyme‐linked immunosorbent assay
Fe‐S: iron‐sulfur cluster
GPI‐CP: glycosylphosphatidylinositol‐linked ceruloplasmin
HAMP: hepcidin antimicrobial peptide (Hepcidin)
H‐Ferritin/FTH1: ferritin heavy chain
ICP‐MS: inductively coupled plasma mass spectrometry
IRE: iron‐responsive element
IRP: iron‐responsive protein
KO: knockout
LAMP1: lysosomal‐associated membrane protein 1
LC3: microtubule associated protein 1 light chain 3
L‐Ferritin/FTL: ferritin light chain
mtDNA: mitochondrial DNA
NBIA: neurodegeneration with brain iron accumulation
NCOA4: nuclear receptor coactivator 4
nDNA: nuclear DNA
NTBI: nontransferrin‐bound iron
OCR: oxygen consumption rate
ROS: reactive oxygen species
RRID: Research Resource Identifier
rWDR45: recombinant WDR45
sCP: secreted ceruloplasmin
SENDA: static encephalopathy of childhood with neurodegeneration in adulthood
SFM: serum‐free medium
SLC11A2/DMT1: solute carrier family 11 member 2/divalent metal transporter 1
SLC39A14/ZIP14: solute carrier family 39 member 14/zinc transporter ZIP14
SLC39A8/ZIP8: solute carrier family 39 member 8/zinc transporter ZIP8
SLC40A1/FPN: solute carrier family 40 member 1/Ferroportin‐1
SQSTM1: sequestosome 1
TBI: transferrin‐bound iron
TdT: terminal deoxynucleotidyl transferase
TF: transferrin
TFRC: transferrin receptor
TUNEL: terminal deoxynucleotidyl transferase‐mediated dUTP‐biotin nick end labeling
WDR45: WD repeat domain phosphoinositide‐interacting protein 4
WT: wild type

## 1 INTRODUCTION

Neurodegeneration with brain iron accumulation (NBIA) is a clinically and genetically heterogeneous group of neurodegenerative diseases characterized by the abnormal accumulation of brain iron and progressive extrapyramidal dysfunction (Levi & Finazzi, 2014). MRI analyses of the brains of patients with NBIA have indicated that the most prominent iron accumulation is in the basal ganglia, particularly in the globus pallidus and the substantia nigra (Haack et al., 2012; Hayflick et al., 2013). Thus far, 15 genes have been identified to be accountable for NBIA (Levi et al., 2019). Only two of the causative genes have been directly associated with mutations in the known iron metabolism proteins, including CP (ceruloplasmin) and FTL (ferritin light chain) (Hayflick et al., 2018). The roles of the remaining genes that participate in iron metabolism have yet to be determined for a variety of cellular processes, such as lipid metabolism, lysosomal activity, and autophagic processes (Arber et al., 2016; Lu et al., 2011; Wang et al., 2019). Currently, no effective treatment is available for NBIA.

β‐propeller protein‐associated neurodegeneration (BPAN; OMIM #300894) is a recently identified subtype of NBIA (Hayflick et al., 2013). Despite the location of the gene on the X chromosome, BPAN does not follow the classic X‐linked dominant inheritance. Females have a higher incidence of BPAN than males (e.g., 20 females vs. 3 males), likely because of the lethality of hemizygous mutation in males (Hayflick et al., 2013). Female patients are all heterozygous. The phenotypes of the affected male patients carrying BPAN mutations are clinically indistinguishable from females (Hayflick et al., 2013). Patients of both genders display a similar phenotype because of somatic or germline mosaicism (Haack et al., 2013). BPAN patients display global developmental delays in infancy and early childhood, followed by neurological deterioration in early adulthood with progressive dystonia, parkinsonism, cognitive decline, and seizures (Haack et al., 2012; Hayflick et al., 2013; Saitsu et al., 2013; Schneider et al., 2013). Recent studies on exome sequencing have identified *de novo* mutations in the *WDR45* (WD repeat domain 45) gene in BPAN patients (Haack et al., 2012; Saitsu et al., 2013). These heterozygous mutations involve both missense and truncation mutations that lead to the loss of WDR45 function (Haack et al., 2012; Saitsu et al., 2013). The upregulation of toxic iron in the fibroblasts of two BPAN‐affected patients (Ingrassia et al., 2017) suggests that alterations in iron homeostasis are associated with WDR45 deficiency. Recent data on fibroblasts and pluripotent stem cell‐derived neurons from a female patient with BPAN have shown elevated iron levels and mitochondrial and lysosomal dysfunction (Seibler, 2018). However, these studies did not directly assess how WDR45 deficiency leads to disruptions in brain iron homeostasis.

WDR45 regulates autophagy, an evolutionarily conserved mechanism of the degradation and recycling of dysfunctional cellular components (Klionsky & Emr, 2000). Structured as a beta‐propeller scaffold protein, WDR45 is one of the four mammalian homologs of yeast Atg18, which plays important roles in regulating autophagosome formation (Bakula et al., 2017). WDR45 has attracted considerable attention recently because of its interaction with ATG2A, a lipid transfer protein that is important for expansion of the phagophore, the precursor to the double‐membrane autophagosome that transports cargo during autophagy (Chowdhury et al., 2018; Maeda et al., 2019). Lymphoblast cells derived from BPAN patients exhibit lower autophagic activity and the accumulation of early autophagic structures (Saitsu et al., 2013), which supports the loss‐of‐function mechanisms underlying BPAN. Rodent models further reveal the critical roles of WDR45 in neuronal development and survival. Neuron‐specific *wdr45*‐knockout (KO) mice display poor motor coordination, learning and memory defects, and extensive axon swelling (Zhao et al., 2015). The primary neuronal cells in these mice show defects in autophagic flux, with the accumulation of ubiquitin‐positive aggregates in both neurons and axons (Zhao et al., 2015). In addition, constitutive *wdr45*‐KO mice display cognitive impairments, abnormal synaptic transmission, and lesions in several brain regions (Wan et al., 2019). The defect in autophagy caused by WDR45 deficiency leads to elevated ER stress and neuronal apoptosis (Seibler, 2018), which is consistent with neurodegeneration found in BPAN patients. However, how the roles of WDR45 in autophagy are related to iron accumulation in BPAN patients remains unknown.

Iron is an essential trace element required by almost all living organisms (Ganz, 2013). As the most metabolically active organ in the body, the brain requires iron for oxidative metabolism, myelination, mitochondrial energy generation, and the biosynthesis of neurotransmitters (Madsen & Gitlin, 2007; Salvador, 2010; Todorich et al., 2008). However, excess iron becomes toxic because of the generation of highly reactive free radicals through Fenton chemistry, resulting in oxidative stress and directly damaging DNA, lipids, and proteins (Uttara et al., 2009). Therefore, the brain requires precise iron homeostasis to guard against iron accumulation while providing the optimal level of this nutrient, which is essential for its development and functions.

Ferritinophagy is one of the mechanisms that could link the roles of WDR45 in autophagy to iron homeostasis. Ferritinophagy is the process through which the intracellular iron storage protein ferritin is sequestered within autophagosomes and delivered to lysosomes for degradation (Mancias et al., 2014). This process is crucial in liberating iron from ferritin and thus in maintaining cellular iron homeostasis (Mancias et al., 2014). Because of the role of WDR45 in autophagy, we hypothesize that WDR45 deficiency promotes defects in ferritinophagy, which may underlie iron accumulation. The molecular mechanisms that link alterations in the autophagy function to changes in iron levels by WDR45 deficiency remain unknown. Thus, the goal of the present study is to define the contribution of WDR45 to iron homeostasis, including iron uptake and ferritinophagy, and to determine the functional consequences of iron accumulation.

## 2 MATERIALS AND METHODS

### 2.1 Generation of WDR45 KO SH‐SY5Y cell lines

The *WDR45* CRISPR‐edited SH‐SY5Y cell lines were generated by electroporation of CRISPR‐Cas9 ctRNPs using the Neon Transfection System (ThermoFisher) (Jacobi et al., 2017). Briefly, tracrRNA and crRNA (target sequence: 5'‐ATGACTCAACAGCCACTTCG‐3') were annealed at equimolar amounts by heating at 95°C and slow cooling at room temperature to form ctRNA complexes. Recombinant spCas9 protein (IDT) and annealed ctRNA were separately diluted in Buffer R (supplied with the Neon Transfection System) to a concentration of 40 μM each. Equal amounts of spCAS9 protein and ctRNA were added together and incubated at room temperature for 20 min to form ctRNPs. SH‐SY5Y cells were trypsinized, washed 3 times with 1X PBS, and 2 × 10^5^ cells were resuspended in 18 μL of Buffer R. Two microliters of ctRNP were added to the 18‐μL aliquot of cells and incubated at room temperature for 5 min. Using a 10‐μl Neon tip, each 10‐μl aliquot of the cell suspension was electroporated using 2 pulses at 1200V and 20‐ms pulse width. Cells were dispensed in separate wells of a 96‐well plate with pre‐warmed media. Cells were allowed to recover for 48 h. Cells were then trypsinized and washed 2 times with 1× PBS. Cells were resuspended in 500 μL of 1× PBS and single‐cell sorted on a MoFlo Astrios Cell Sorter (Beckman Coulter, University of Michigan Flow Core). Genomic DNAs from clonal cell lines were isolated using the Puregene Cell and Tissue Culture Kit (Qiagen). PCR products containing the predicted CRISPR cut site were amplified (WDR45‐F1: 5'‐GCAGTCACTCCAGAGTCAGTAGTTAG; WDR45‐R1: 5'‐GTAGATGCGCACACCTGTCTCCATG) and sequenced (University of Michigan DNA Sequencing Core).

### 2.2 Cell culture and reagents

All culture media and supplements were purchased from Invitrogen. Heat‐inactivated fetal bovine serum was purchased from Sigma–Aldrich. SH‐SY5Y human neuroblastoma cells (RRID:CVCL_0019) were grown in DMEM containing 10% fetal bovine serum, penicillin (100 IU/ml), and streptomycin (100 mg/ml) at 37°C in a humidified, 5% CO_2_ incubator. This cell line is not listed in the International Cell Line Authentication Committee (ICLAC) database of cross‐contaminated or misidentified cell lines. No further authentication was performed in the laboratory. All the cells we used in this study were within 20 passages. The MYC‐FLAG‐tagged human *WDR45* plasmid was purchased from Origene (RC209654). The dual‐tagged mCherry‐GFP‐LC3 plasmid was procured from Addgene (123230; deposited by Robin Ketteler). For small interfering RNA (siRNA)‐mediated gene suppression of ferritin, SH‐SY5Y cells were plated in six‐well plates overnight and then transfected with 50 nM of control siRNA (SIC001‐10NMOL; MilliporeSigma) or *FTH1* siRNA (SASI_Hs01_00112824) using Lipofectamine 3000 (Thermo Fisher Scientific) according to the manufacturer's specifications. Pyrimidinone 8 was purchased from Sigma–Aldrich (ENA432539789).

### 2.3 TF‐bound and Non‐TF‐bound ^59^Fe uptake assays

For TF‐bound ^59^Fe uptake assays, the loading of ^59^Fe onto TF was performed as previously described (Burdo et al., 2003). Briefly, ^59^FeCl_3_ (PerkinElmer Life Sciences) was incubated with TF‐loading buffer (0.1 M HEPES, pH 7.5, 0.15 M NaCl) containing 20 mM NaHCO_3_ and 88 μM nitrilotriacetic acid for 5 min at room temperature. Then, 20 μM apo‐TF was added and incubated for 2 h at RT. Non‐TF‐bound ^59^Fe was removed from the ^59^Fe‐TF solution by buffer exchange using a Nanosept 10K molecular weight cutoff Omega spin column (PALL Corporation) and spinning 3 × 10 min at 2841 g. This procedure yielded a ^59^Fe‐TF purity of >95%. Cells were washed three times with prewarmed PBS and incubated for 90 min at 37°C with serum‐free medium containing ^59^Fe‐TF. Cells were chilled on ice and washed twice with ice‐cold PBS. Radioactivity was determined with a gamma counter and normalized to the cell protein measured in lysates using the Bradford assay. A non‐TF–bound ^59^Fe uptake assay was performed as described previously (Choi et al., 2019). Cells were washed twice with serum‐free medium (SFM) and incubated for 1 h in SFM containing 2% bovine serum albumin to bind residual TF and prevent iron uptake via TF‐bound iron endocytosis. After incubation, cells were washed with serum‐free medium and incubated with 1 μM ^59^Fe with 150 μM ascorbate in pH 6.0 or 7.4 uptake buffer at 37°C. Cells were chilled on ice for 5 min, washed three times with ice‐cold PBS containing iron chelator solution (1 mM bathophenanthroline sulfonate and 1 mM diethylenetriaminepentaacetic acid) to remove any residual ^59^Fe, and then were directly harvested for intracellular radioactivity. Radioactivity was determined with a gamma counter and normalized to the cell protein measured in lysates using the Bradford assay.

### 2.4 Trace element analysis

Cells were grown on 100‐mm tissue culture dishes and analyzed for metals by inductively coupled plasma mass spectrometry (ICP‐MS) as we described previously (Choi et al., 2018, 2019, 2020). Briefly, the cell samples were digested with 2 ml/g total wet weight nitric acid (BDH ARISTAR^®^ULTRA) for 24 h and then digested with 1 ml/g total wet weight hydrogen peroxide (BDH Aristar^®^ ULTRA) for 24 h at room temperature. The samples were stored at 4°C until metals were quantified. Ultrapure water was used for final sample dilution. For mitochondrial iron levels, mitochondria were isolated through differential centrifugation as we previously described (Choi et al., 2018), and then mitochondrial iron levels were measured via ICP‐MS.

### 2.5 RNA isolation and qPCR

Total RNA was isolated from cells using TRIzol reagent (Invitrogen) following the manufacturer's instructions. Purified RNA was then reverse‐transcribed with SuperScript^®^ III First‐Strand Synthesis System (Invitrogen). The qRT‐PCR was performed using Power SYBR‐Green PCR Master Mix (Applied Biosystems). *18S RNA* was used for normalization of the mRNA. The primers used for qPCR are listed in Table S1 and were all purchased from Integrated DNA Technologies.

### 2.6 MtDNA copy number

Total DNA was extracted from cell samples via TRIzol (Invitrogen) extraction as described previously (Choi et al., 2018). Following complete removal of the RNA‐containing aqueous phase, DNA extraction buffer [Tris base (1 M), sodium citrate dibasic trihydrate (50 mM), and guanidine thiocyanate (4 M)] were added to the tubes containing the remaining Trizol‐separated interphase and infranatant. The tubes were shaken vigorously and centrifuged at 12 000 *g* at room temperature for 30 min. The aqueous phase was collected, and the genomic and mitochondrial DNA was precipitated in isopropanol. Samples were respun at 12 000 *g* at 4°C to pellet the DNA. The DNA pellet was then washed in 70% ethanol, respun, and, after careful ethanol removal, resuspended in TE buffer. To quantify the mtDNA copy number, qPCR was performed as described above against external standards for mtDNA and β‐globin using primers listed in Table S1.

### 2.7 Immunoblot analysis

The total lysates were prepared in RIPA buffer plus protease inhibitors (Roche, 11836153001). Protein concentrations were determined by Bradford assay. Samples were separated by electrophoresis and transferred to a nitrocellulose membrane (Bio‐Rad, 1620115). The membrane was immunoblotted with anti‐mouse FTL/ferritin light chain antibody (Santa Cruz Biotechnology, sc‐74513), anti‐rabbit FTH/ferritin heavy chain antibody (Cell Signaling Technology, 4393S), anti‐rabbit total ferritin antibody (Sigma‐Aldrich, F5012), anti‐rabbit LC3B antibody (Cell Signaling Technology, 2775), anti‐rabbit SQSTM1/p62 (Cell Signaling Technology, 5114), anti‐rabbit WDR45 antibody (ProteinTech, 19194‐1‐AP), anti‐rabbit flag antibody (Sigma‐Aldrich, F7425), anti‐rabbit NCOA4 antibody (Bethyl Laboratories, A302272A), anti‐mouse CYCS/cytochrome *c* antibody (Santa Cruz Biotechnology, sc‐13156), and anti‐mouse ACTB/actin (Proteintech, 60008‐1‐Ig). The blots were visualized with infrared anti‐mouse or anti‐rabbit secondary antibodies, using a LI‐COR Odyssey fluorescent western blotting system (LI‐COR Biosciences). Protein expression was quantified using densitometry (Image Studio Lite; LI‐COR).

### 2.8 Immunofluorescence and microscopy

For confocal studies, cells were plated onto glass coverslips and fixed with 4% paraformaldehyde in PBS for 10 min, and immunofluorescence staining was performed as previously described (Choi et al., 2018, 2019, 2020). To permeabilize the cells, cells were incubated with 0.2% Triton X‐100 in PBS for 5 min. Nonspecific binding was blocked with 4% bovine serum albumin in PBS for 30 min, and cells were detected after incubation with anti‐mouse ferritin (Santa Cruz Biotechnology, sc‐74513) for 1 h. Detection of ferritin was performed by using an anti‐mouse IgG antibody conjugated to Alexa 488 (Thermo Fisher Scientific, A11029) for 20 min. Rabbit anti‐LC3 (Cell Signaling Technology, 2775) or rabbit anti‐LAMP1 (Proteintech, 21997‐1‐AP) was used as a marker of autophagosomes or lysosomes, respectively. Detection of LC3 or LAMP1 was performed by using an anti‐rabbit IgG antibody conjugated to Alexa Fluor 568 (Thermo Fisher Scientific, A11036) for 20 min. Coverslips were drained, mounted in ProLong Gold (Thermo Fisher Scientific), and sealed with nail polish. Immunofluorescence imaging was performed by using a Nikon Eclipse A‐1 confocal microscope (Nikon Instruments) with an X60 oil immersion lens. For the dual fluorescent LC3 assay, cells were transfected with dual tagged mCherry‐GFP‐LC3 and were then fixed 48h post transfection. Green vs. green/red (yellow) vesicles were quantified by analyzing 20 different images by two individuals, one blind to the experimental design and data analysis. Exposure settings were unchanged throughout acquisition.

### 2.9 Calcein‐AM assays for labile iron pool

Cells were grown in a black walled 96‐well plate and incubated overnight. Medium was removed, and cells were washed three times with PBS and then incubated with 1 µM calcein‐AM (Life Technologies) in PBS for 20 min at 37°C in 5% CO_2_. Cell‐associated fluorescence (excitation, 488 nm; emission, 517 nm) was measured at 25°C using a plate reader. Reduced fluorescence intensity reflects quenching as a result of free iron binding to calcein.

### 2.10 Measurement of ROS formation

ROS levels were measured using a fluorescent indicator specific for H_2_O_2_ production (H_2_DCFDA) as described previously (Choi et al., 2018, 2019). H_2_DCFDA, a cell‐permeant ROS indicator, is nonfluorescent and can be converted to highly fluorescent 2',7'‐dichlorofluorescein (DCF) following the removal of the acetate groups by intracellular esterases and ROS‐induced oxidation. Cells were incubated with 3 μM DCFH‐DA (Invitrogen) for 30 min at 37°C. Fluorescence of DCF (excitation 495 nm, emission 520 nm) was measured at 25°C using a plate reader.

### 2.11 Seahorse XFe96 extracellular flux analysis

Extracellular flux analyses were performed using a Seahorse XFe‐96 analyzer (Agilent Technologies), according to the manufacturer's instructions. Twenty‐four hour before the assay, cells were cultured on Seahorse XF‐96 plates at a density of 3 × 10^4^ cells per well. Cells were washed and incubated with XF assay Medium (Seahorse Bioscience), supplemented with 25 mM glucose, 1 mM sodium pyruvate, 2 mM l‐glutamine at 37°C and 0% CO_2_ for 1 h. The baseline oxygen consumption rates (OCR) were measured at 37°C four times before sequentially injecting the following: Oligomycin (2 μM) to measure the ATP‐linked OCR, the oxidative phosphorylation uncoupler FCCP (1.5 μM) to determine maximal respiration, and rotenone (1 µM) and antimycin A (1 µM) to determine the non‐mitochondrial respiration. The baseline extracellular acidification rates (ECAR) were measured at 37°C four times before sequentially injecting the following: d‐glucose (10 mM) to measure the glycolytic rate, oligomycin (2 μM) to inhibits the mitochondrial ATP synthase, and 2‐deoxy‐d‐glucose (2‐DG; 50 mM) to inhibit glycolysis. OCR and ECAR were automatically calculated by the Seahorse XFe‐96 software.

### 2.12 Isoprostane analysis

Isoprostane was chosen as a marker of oxidative damage and measured by a competitive enzyme‐linked immunosorbent assay (ELISA) for one of the isoprostanes 8‐iso Prostaglandin F2α (8‐iso‐PGF) with a commercial kit (Cayman Chemical). The assay was based on the competition between 8‐iso‐PGF and 8‐isoprostane‐acetylcholinesterase (AChE) conjugate for a limited number of binding sites in each ELISA plate well. The concentration of 8‐iso‐PGF is inversely proportional to the number of binding sites available, whereas AChE is held constant. The assays were performed as we described previously (Choi et al., 2018, 2020).

### 2.13 CASP3 activity

The CASP3 activity in cells was measured by the Caspase‐3 Assay Kit (Abcam, ab39401). Briefly, the cells were harvested and lysed on ice. After centrifugation, protein was measured and then adjusted to the concentration as suggested, then the CASP3 activity was examined by following the manufacturer's instructions.

### 2.14 Proliferation assay

Cells were plated on 96‐well plates. After 2 days of cell growth, 10 µL/well of WST‐8 reagent (cell counting kit 8; Sigma‐Aldrich, 96992) was added and absorbance at 450 nm was measured by using a BioTek Synergy microplate reader (BioTek Instruments). Medium without cells was used as background, and the A_450_ of background was subtracted from the samples.

### 2.15 TUNEL assay

Apoptosis was quantified using the terminal deoxynucleotidyltransferase‐mediated dUTP‐biotin nick end labeling (TUNEL) assay using In Situ Cell Death Detection Kit, TMR‐Red (Roche, 12156792910). In brief, cells were fixed with 4% paraformaldehyde in PBS for 1 h at room temperature and were permeabilized with 0.1% Triton X‐100 in 0.1% sodium citrate. After an extensive wash in PBS, specimens were incubated with a solution containing terminal deoxynucleotidyl transferase (TdT) and TMR red‐labeled dUTP for 1 h at 37°C. All the samples were mounted in ProLong Gold (Thermo Fisher Scientific). Apoptotic cells were visualized under fluorescence microscopy. Chromatin was stained with 4',6‐diamidino‐2‐phenylindole (DAPI) (Thermo Fisher Scientific, D1306), and nuclei were counted to quantify the number of cells in each field. A minimum of 500 cells in an average of 15 fields were counted on each slide. Cells undergoing apoptosis were counted only when they stained positively in the TUNEL assay and displayed a pyknotic nucleus, as determined by DAPI staining. All the slides were quantified by analyzing 20 different images by two individuals, one blind to the experimental design and data analysis.

### 2.16 Study design and statistical analysis

This study was not pre‐registered. Institutional ethics approval was not required for our study. No randomization was performed to allocate treatments in the study. No exclusion criteria were predetermined. Statistical analysis was performed using GraphPad Prism 8 (GraphPhad Software).

Data are presented as individual values and represent the means ± SEM. To compare two groups, two‐tailed *p* values were calculated using an unpaired *t*‐test. To compare more than two groups, *p* values were calculated using 1‐way ANOVA with Tukey's multiple comparisons test or two‐way ANOVA with Bonferroni's multiple comparisons test as specified in each figure legend. Normality of the data was not assessed. Sample sizes were not statistically pre‐determined but were estimated based on previous studies (Choi et al., 2018, 2019) and similar to those generally used in the field. Outliers were identified using the GraphPad ROUT (robust regression and outlier removal) method (*Q* = 1%). Values of *p* < 0.05 were considered statistically significant. Asterisks in graphs, wherever present, denote statistically significant differences.

## 3 RESULTS

### 3.1 Generation of a *WDR45*‐KO SH‐SY5Y cell line

To assess the role of WDR45 in neuron‐related cells, we inactivated the *WDR45* gene in SH‐SY5Y neuroblastoma cells (SH‐SY5Y_ΔWDR45_). The SH‐SY5Y cell line maintains many neuronal properties and has been used to investigate other neurodegenerative diseases (Xicoy et al., 2017). We used CRISPR‐Cas9 to target editing near the start codon in exon 3 of *WDR45* (Figure S1a). A CRISPR‐Cas9‐mediated cut introduced a homozygous 64‐bp deletion, which spanned exon 3 and intron 3 (Figure S1b,c). The resulting RNA is predicted to encode a 58 amino acid polypeptide with a translation start in exon 3 and a premature stop in intron 3 (data not shown). Quantitative PCR (qPCR) analysis, using primer pairs that amplified a downstream segment of *WDR45* mRNA (exons 8–9), showed an ~84% reduction compared with the wild type (WT) (Figure S1d), indicating that the mutant *WDR45* mRNA underwent nonsense‐mediated decay. These results confirmed the inactivation of the *WDR45* gene in SH‐SY5Y_Δ_ *_WDR45_* cells.

### 3.2 Loss of WDR45 enhances intracellular iron levels

**FIGURE 1:**
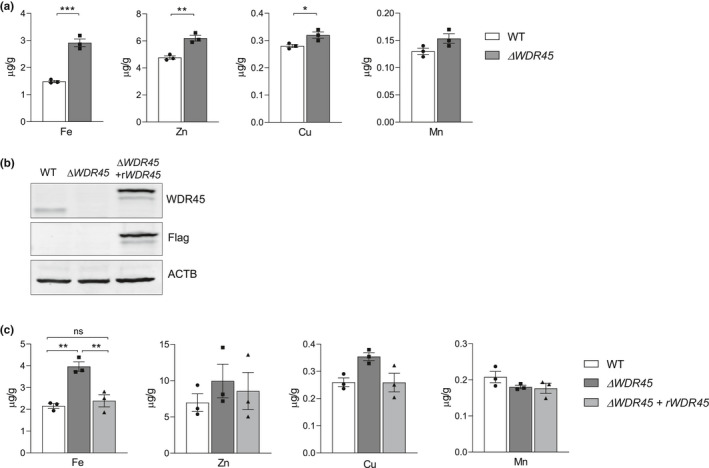
Elevated levels of iron in SH‐SY5Y_Δ_ *_WDR45_* cells. (a) Iron (Fe), zinc (Zn), copper (Cu), and manganese (Mn) levels were measured by ICP‐MS. *n* = 3 independent cell culture preparations. Student's *t*‐test (*p < 0.05, **p < 0.01, and ***p < 0.001). (b) Representative immunoblot of WDR45 levels in total cell lysates isolated from SH‐SY5Y_WT_ cells, SH‐SY5Y_Δ_ *_WDR45_* cells, and SH‐SY5Y_Δ_ *_WDR45_* cells expressing recombinant *WDR45* (r*WDR45*). Equal loading was verified by immunoblotting with ACTB/actin antibody. (c) Metal levels in SH‐SY5Y_WT_ and SH‐SY5Y_Δ_ *_WDR45_* cells, and SH‐SY5Y_Δ_ *_WDR45_* cells expressing rWDR45. *n* = 3 independent cell culture preparations. *n* = 3 independent cell culture preparations. One‐way ANOVA followed by post hoc Tukey's multiple comparisons test (*p < 0.05 and **p < 0.01)

Brain iron accumulation is a key hallmark among the heterogeneous group of NBIA, including BPAN (Levi et al., 2019). Thus, we used inductively coupled plasma mass spectrometry (ICP‐MS) to measure the total content of metals in SH‐SY5Y_Δ_ *_WDR45_* cells. The total iron content was significantly increased ~2.0‐fold (*t*‐test, *t* = 9.778, df = 4, *p* = 0.0006) in SH‐SY5Y_Δ_ *_WDR45_* cells compared with the WT SH‐SY5Y (SH‐SY5Y_WT_) cells (Figure 1a). We also measured the levels of other trace elements, including zinc, copper, and manganese. Unexpectedly, a significant increase in the total zinc and copper content (Zn ~1.3‐fold; *t*‐test, *t* = 5.963, df = 4, *p* = 0.0040, Cu ~1.1‐fold; *t*‐test, *t* = 3.098, df = 4, *p* = 0.0363), although weaker than that of iron, was detected in SH‐SY5Y_Δ_ *_WDR45_* cells (Figure 1a). In contrast, the levels of manganese did not differ between the genotypes (Figure 1a). No heavy metals, such as cadmium and lead, were detected in these samples.

To confirm the role of WDR45 in iron homeostasis, we next performed cDNA complementation assays, in which we introduced a MYC‐FLAG‐tagged human *WDR45* cDNA (rWDR45; see Materials and Methods) into SH‐SY5Y_Δ_ *_WDR45_* cells. Immunoblot analysis using an anti‐WDR45 antibody showed SH‐SY5Y_WT_ cells, but not SH‐SY5Y_Δ_ *_WDR45_* cells, expressed endogenous WDR45 at ~35 kDa, further verifying inactivation of WDR45 in these KO cells (Figure 1b, top panel). SH‐SY5Y_Δ_ *_WDR45_* cells expressing recombinant WDR45 (rWDR45) had a higher band of WDR45 at ~40 kDa (Figure 1b, top and middle panel) detected by both anti‐WDR45 and anti‐FLAG antibodies; this shift in migration was likely because of the MYC‐FLAG epitope tags that were introduced into the recombinant construct. We then measured metal levels by ICP‐MS. Expression of recombinant WDR45 in SH‐SY5Y_Δ_ *_WDR45_* cells restored the iron concentration to levels similar to those of SH‐SY5Y_WT_ cells (Figure 1c, One‐way ANOVA, *F*(2, 6) = 21.39, *p* = 0.0019). The reintroduction of WDR45 did not significantly influence the levels of zinc, copper, and manganese (Figure 1c). Thus, these data demonstrate a direct link between the expression of WDR45 and intracellular iron levels.

### 3.3 Loss of WDR45 alters cellular iron acquisition pathways

**FIGURE 2:**
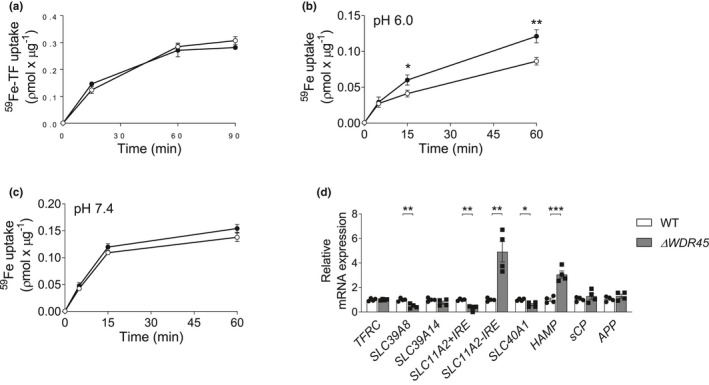
Uptake assays of ^59^Fe and expression of iron‐related factors in SH‐SY5Y_Δ_ *_WDR45_* cells. (a) Cells were incubated with ^59^Fe‐TBI in uptake buffer at 37°C. (b and c) Cells were incubated with ^59^Fe‐NTBI in pH 6.0 and 7.4 uptake buffer at 37°C. Whole cell ^59^Fe content was determined at the indicated time points using a γ‐counter. Each sample was normalized for protein concentration. *n* = 3 independent cell culture preparations. Two‐way ANOVA followed by post hoc Bonferroni's multiple comparisons test (*p < 0.005 and **p < 0.0001). (d) Relative transcription levels of iron‐related proteins were detected using qPCR. *n* = 4 independent cell culture preparations. Student's *t*‐test (*p < 0.05, **p < 0.01, and ***p < 0.001)

To identify the mechanism by which the loss of *WDR45* increases total iron levels, we examined the cellular iron uptake pathways. Cellular iron acquisition involves two distinct pathways: (1) the canonical TF (transferrin)‐bound iron (TBI) uptake pathway and (2) the nontransferrin‐bound iron uptake (NTBI) pathway. We first examined the kinetics of the uptake of ^59^Fe by the TBI pathway in SH‐SY5Y_WT_ and SH‐SY5Y_Δ_ *_WDR45_* cells. The cells were incubated with ^59^Fe‐TF at 37°C, and then cell‐associated ^59^Fe was monitored over time. We found that ^59^Fe‐TBI uptake kinetics did not differ between the two groups (Figure 2a). We then examined the kinetics of ^59^Fe‐NTBI uptake by SH‐SY5Y_Δ_ *_WDR45_* cells. The major NTBI transporters, SLC11A2/DMT1 (solute carrier family 11 member 2) and SLC39A8/ZIP8‐SLC39A14/ZIP14, showed distinct pH activities. SLC11A2/DMT1 was the most efficient at pH 5.5–6.0, whereas SLC39A8/ZIP8 and SLC39A14/ZIP14 showed maximal iron transport near or higher than the physiological pH of 7.0 (Knutson, 2017; Wang et al., 2012). Thus, we examined ^59^Fe‐NTBI uptake under different pH conditions. Notably, the uptake of ^59^Fe‐NTBI at pH 6.0 was significantly higher in SH‐SY5Y_Δ_ *_WDR45_* cells than in SH‐SY5Y_WT_ cells (Figure 2b, Two‐way ANOVA, *F*(3, 12) = 13.70, *p* = 0.0004). At pH 7.4, ^59^Fe‐NTBI uptake did not differ significantly between the two groups (Figure 2c). These results suggest that the NTBI uptake driven by SLC11A2/DMT1 was aberrantly enhanced in SH‐SY5Y_Δ_ *_WDR45_* cells.

### 3.4 Altered expression of iron‐related proteins in SH‐SY5Y_Δ_ *_WDR45_* cells

To further corroborate the contribution of a specific iron transporter, we examined the mRNA expression of known iron transporters involved with iron uptake and efflux within the brain. The iron transporters that we monitored included TFRC (transferrin receptor), SLC39A8/ZIP8, SLC39A14/ZIP14, SLC11A2/DMT1, and SLC40A1/FPN/ferroportin (Bogdan et al., 2016). Iron‐responsive elements (IREs) in *TFRC* and an isoform of *SLC11A2*/*DMT1*, *SLC11A2*/*DMT1*+IRE, contribute to cellular iron homeostasis. When the cytosolic iron level increases, iron‐responsive proteins (IRPs) bind to IREs, which reside in the 5’ and 3’ untranslated regions of the *TFRC* and *SLC11A2*/*DMT1*+IRE mRNAs. The IRP–IRE interaction increases the degradation of these mRNAs, which limits the iron uptake into the cells. However, the *SLC11A2*/*DMT1*−IRE isoform is not influenced by intracellular iron levels, although it can be upregulated at the transcriptional (Paradkar & Roth, 2006) or post‐translational level (Garrick et al., 2012). Our qPCR analysis showed that SH‐SY5Y_Δ_ *_WDR45_* cells displayed significantly increased *SLC11A2*/*DMT1*−IRE transcript levels (Figure 2d). In contrast, *SLC39A8*/*ZIP8*, *SLC11A2*/*DMT1*+IRE, and *SLC40A1*/*FPN* transcript levels were significantly lower in SH‐SY5Y_Δ_ *_WDR45_* cells than in SH‐SY5Y_WT_ cells. No significant differences in the *TFRC* and *SLC39A14*/*ZIP14* transcript levels were detected (Figure 2d). We also measured the mRNA expression of *HAMP* (hepcidin antimicrobial peptide), a hormone peptide that inhibits iron transport through the internalization and degradation of SLC40A1/FPN (Nemeth et al., 2004). The *HAMP* transcript level was significantly higher in SH‐SY5Y_Δ_ *_WDR45_* cells than in SH‐SY5Y_WT_ cells (Figure 2d). We further measured the mRNA expression of iron‐related proteins, including CP (ceruloplasmin) and APP (amyloid beta precursor protein). CP is the iron ferroxidase that facilitates SLC40A1/ferroportin export activity and is widely expressed throughout the brain (Hadziahmetovic et al., 2008). Two molecular isoforms of CP exist: secreted CP (sCP) and a membrane glycosylphosphatidylinositol (GPI)‐anchored form of CP (GPI‐CP). In the brain, sCP is detectable in the cerebrospinal fluid (Paradowski et al., 1995), whereas GPI‐CP is expressed in mammalian astrocytes (Patel & David, 1997). No significant difference was detected in the level of the transcript encoding sCP between SH‐SY5Y_WT_ and SH‐SY5Y_Δ_ *_WDR45_* cells (Figure 2d). In our hands, the transcript encoding GPI‐CP was not detected in both groups. APP is another iron‐related protein known to have a functional role in iron homeostasis through stabilizing the iron efflux protein SLC40A1/FPN (Duce et al., 2010; McCarthy et al., 2014; Rogers et al., 2016; Wong et al., 2014). We also did not detect any significant difference in the *APP* transcript level between SH‐SY5Y_WT_ and SH‐SY5Y_Δ_ *_WDR45_* cells (Figure 2d). The results of *t*‐test are as follows: *SLC39A8*/*ZIP8*; *t* = 4.873, df = 6, *p* = 0.0028, *SLC11A2*/*DMT1*+IRE; *t* = 5.902, df = 6, *p* = 0.0011, *SLC11A2*/*DMT1*−IRE; *t* = 4.722, df = 6, *p* = 0.0033, *SLC40A1*/*FPN*; *t* = 3.628, df = 6, *p* = 0.0110, *HAMP*; *t* = 6.397, df = 6, *p* = 0.0007 (Figure 2d). Taken together, these data suggest that the loss of WDR45 leads to the dysregulation of iron transporter genes and that the upregulation of iron uptake transporter *SLC11A2*/*DMT1*−IRE expression and the downregulation of the iron exporter gene *SLC40A1*/*FPN* may underlie the acquisition of NTBI in SH‐SY5Y_Δ_ *_WDR45_* cells. The reduction of the IRE‐containing mRNAs, *TFRC* and *SLC11A2*/*DMT1*+IRE, may be the consequence of iron accumulation within SH‐SY5Y_Δ_ *_WDR45_* cells.

### 3.5 Autophagic flux in SH‐SY5Y_Δ_ *_WDR45_* cells

Previous studies on *WDR45* mutant patient‐derived lymphoblast cells and *wdr45*‐KO mice have shown defective autophagic flux (i.e., the rate of autophagy) (Saitsu et al., 2013; Wan et al., 2019; Zhao et al., 2015). Thus, we sought to determine whether our SH‐SY5Y_Δ_ *_WDR45_* cells also exhibited altered autophagic flux. During autophagy, a cytosolic proteolytically processed form of MAP1LC3/LC3 (microtubule‐associated protein 1 light chain 3), referred to as LC3‐I, is conjugated to phosphatidylethanolamine to form LC3‐II, which in turn is recruited to phagophore membranes (Tanida et al., 2008). When autophagosomes fuse with lysosomes to form autolysosomes, the population of LC3‐II that was present inside the autophagosomes is consequently degraded by lysosomal hydrolases (Mizushima & Yoshimori, 2007; Tanida et al., 2005). Lysosomal dysfunction induced by lysosomal inhibitors leads to the marked accumulation of LC3‐II in autophagic structures (Tanida et al., 2005). Thus, we evaluated the autophagic flux by measuring LC3‐II levels in the absence and presence of the lysosomal inhibitor chloroquine (CQ) (Mauthe et al., 2018; Redmann et al., 2017). Immunoblot analysis revealed no difference in LC3‐II levels between SH‐SY5Y_WT_ and SH‐SY5Y_Δ_ *_WDR45_* cells under basal conditions (i.e., without Torin 1 treatment), although the total amount of LC3 (LC3‐I + LC3‐II) was lower in the SH‐SY5Y_Δ_ *_WDR45_* cells (Figure S2a,b). The induction of autophagy with the MTORC1 inhibitor Torin 1 led to an elevation of LC3‐II levels in SH‐SY5Y_WT_ cells and SH‐SY5Y_Δ_ *_WDR45_* cells (Figure S2a,b). The concomitant treatment with CQ to block autophagosome–lysosome fusion led to a further elevation of the LC3‐II levels in both SH‐SY5Y_WT_ and SH‐SY5Y_Δ_ *_WDR45_* cells, indicating that the Torin 1‐dependent increase in LC3‐II was not the result of a block in fusion (Figure S2a,b). We further assessed the autophagic flux by measuring the endogenous autophagic substrate SQSTM1/p62 (sequestosome 1) levels. SQSTM1/p62, the first autophagic cargo receptor identified in mammalian cells, mediates the formation and autophagic clearance of intracellular protein aggregates (Bjorkoy et al., 2005; Komatsu et al., 2007; Pankiv et al., 2007). Consistent with LC3‐II levels in SH‐SY5Y_WT_ cells, the SQSTM1/p62 levels were reduced by Torin 1 but elevated by the concomitant treatment with CQ (Figure S2a,c). However, in SH‐SY5Y_Δ_ *_WDR45_* cells, the SQSTM1/p62 levels were slightly reduced by Torin 1, but not further changed by the concomitant treatment with CQ; the latter result suggests a partial impairment of degradation in the SH‐SY5Y_Δ_ *_WDR45_* cells. Taken together, these data suggest no significant difference in autophagic flux between SH‐SY5Y_WT_ and SH‐SY5Y_Δ_ *_WDR45_* cells. Instead, the basal levels of autophagy machinery are reduced in SH‐SY5Y_Δ_ *_WDR45_* cells, suggesting the impaired biogenesis of autophagosomes.

### 3.6 Loss of WDR45 impairs ferritinophagy

**FIGURE 3:**
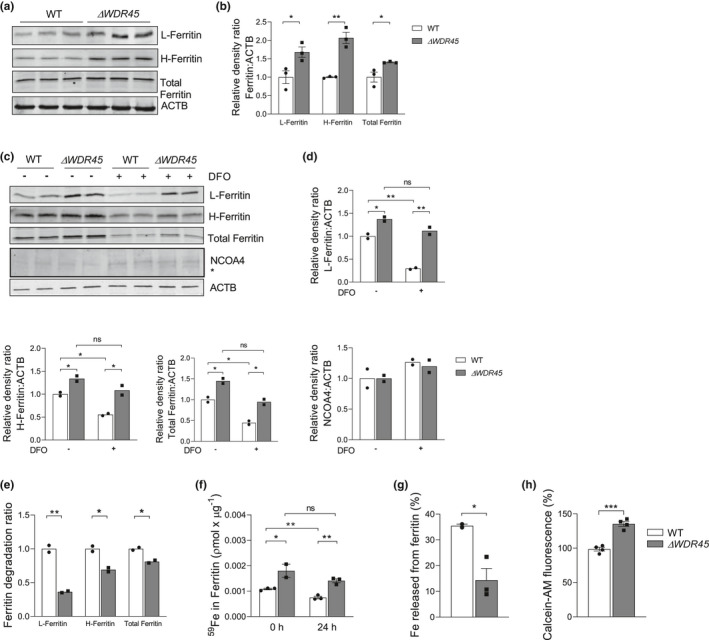
Impaired ferritinophagy in SH‐SY5Y_Δ_ *_WDR45_* cells. (a) Representative immunoblot of L‐ferritin, H‐ferritin, and total ferritin levels in total cell lysates isolated from SH‐SY5Y_WT_ and SH‐SY5Y_Δ_ *_WDR45_* cells. Equal loading was verified by immunoblotting with ACTB/actin antibody. (b) Quantification of L‐ferritin, H‐ferritin, and total ferritin relative protein after normalization with ACTB. *n* = 3 independent cell culture preparations. (c) Representative immunoblot of L‐ferritin, H‐ferritin, total ferritin, and NCOA4 levels in total cell lysates isolated from SH‐SY5Y_WT_ and SH‐SY5Y_Δ_ *_WDR45_* cells treated with or without DFO. Ferritin degradation was stimulated with the iron chelator DFO (100 μm) for 6 h, followed by cell collection and sample preparation. Student's *t*‐test (*p < 0.05, **p < 0.01, and ***p < 0.001). (d) Quantification of L‐ferritin, H‐ferritin, total ferritin, and NCOA4 relative protein after normalization with ACTB. Two‐way ANOVA followed by post hoc Bonferroni's multiple comparisons test (*p < 0.05 and **p < 0.01). (e) The degradation ratio of L‐ferritin, H‐ferritin, and total ferritin with (+) or without (−) DFO treatment. Student's *t*‐test (*p < 0.05 and **p < 0.01). (f) ^59^Fe release from ferritin. Cells were pulsed with ^59^FeCl_3_ for 4 h and chased for 0 h and 24 h. Immunoprecipitation of ferritin was performed, and the ^59^Fe‐ferritin levels in the agarose pellet were determined using a γ‐counter. Two‐way ANOVA followed by post hoc Bonferroni's multiple comparisons test (*p < 0.05 and **p < 0.01). (g) Percentage of Fe released from ferritin during the 24‐h chase period. *n* = 2–3 independent cell culture preparations; Two‐way ANOVA followed by post hoc Bonferroni's multiple comparisons test (*p < 0.05 and **p < 0.01). (h) Calcein‐AM fluorescence in SH‐SY5Y_WT_ or SH‐SY5Y_Δ_ *_WDR45_* cells. *n* = 4 independent cell culture preparations. Student's *t*‐test (***p < 0.001)

Increased intracellular iron is typically sequestered in the iron storage protein ferritin in a soluble and non‐toxic state when it is not immediately required (Anderson & Frazer, 2017). Immunoblot analysis revealed that levels of L‐ferritin, H‐ferritin, and total ferritin were significantly increased ~1.7‐fold (*t*‐test, *t* = 3.031, df = 4, *p* = 0.0387), ~2‐fold (*t*‐test, *t* = 6.888, df = 4, *p* = 0.0023), and ~1.4‐fold (*t*‐test, *t* = 2.939, df = 4, *p* = 0.0424), respectively, in SH‐SY5Y_Δ_ *_WDR45_* cells compared with SH‐SY5Y_WT_ cells (Figure 3a,b). The increased ferritin levels in SH‐SY5Y_Δ_ *_WDR45_* cells prompted us to assess the ferritinophagy process, by which ferritin is sequestered within autophagosomes and delivered to lysosomes for degradation (Mancias et al., 2014; Masaldan et al., 2018). This process is important in liberating iron from ferritin and thus in maintaining cellular iron homeostasis (Mancias et al., 2014). We hypothesized that impaired ferritin degradation explains the iron accumulation phenotype of SH‐SY5Y_Δ_ *_WDR45_* cells. To test this hypothesis, we stimulated ferritin degradation by the iron chelator deferoxamine (DFO). In SH‐SY5Y_WT_ cells, 6‐h DFO treatment led to the substantially increased degradation of L‐ferritin, H‐ferritin, and total ferritin (Figure 3c–e). The results of two‐way ANOVA tests are as follows: L‐Ferrtin/ACTB; *F*(1, 4) = 17.98, *p* = 0.0133, H‐Ferritin/ACTB; *F*(1, 4) = 2.142, *p* = 0.2171, Total‐Ferritin/ACTB; *F*(1, 4) = 0.1859, *p* = 0.6886, NCOA4/ACTB; *F*(1, 4) = 0.1140, *p* = 0.7526 (Figure 3d). In contrast, the DFO treatment did not induce ferritin degradation in SH‐SY5Y_Δ_ *_WDR45_* cells as effectively as it did in the WT (Figure 3c–e). The results of *t*‐test are as follows: L‐Ferrtin; *t* = 12.14, df = 2, *p* = 0.0067, H‐Ferritin; *t* = 6.200, df = 2, *p* = 0.0250, Total‐Ferritin; *t* = 6.718, df = 2, *p* = 0.0215 (Figure 3e). We further examined whether loss of WDR45 function alters NCOA4 (nuclear receptor coactivator 4) levels. NCOA4 has been identified as a cargo receptor targeting ferritin to the lysosome for subsequent degradation (Mancias et al., 2014). Regardless of DFO treatment, no significant changes were detected between SH‐SY5Y_WT_ and SH‐SY5Y_Δ_ *_WDR45_* cells (Figure 3c,d). These results indicate that the loss of WDR45 impaired ferritin degradation without affecting the level of the NCOA4 receptor.

Higher ferritin levels can result in the sequestration of bioactive iron that is trapped by ferritin. To test this possibility, we next conducted a pulse‐chase experiment to monitor the release of iron from ferritin in SH‐SY5Y_Δ_ *_WDR45_* cells, a process that requires the degradation of ferritin (Asano et al., 2011). Equal numbers of SH‐SY5Y_WT_ and SH‐SY5Y_Δ_ *_WDR45_* cells were radiolabeled with equal counts of ^59^Fe for 4 h, followed by a wash‐out and chase in normal medium for 24 h. Ferritin was immunoprecipitated from the cell lysates, and the ferritin‐associated radioactive levels were determined using a gamma counter. As expected, the ^59^Fe levels in the immunoprecipitated ferritin were significantly higher in SH‐SY5Y_Δ_ *_WDR45_* cells than in SH‐SY5Y_WT_ cells immediately after the pulse (Figure 3f). Following a chase of 24 h, the amount of ferritin‐bound iron decreased, but was still significantly higher in SH‐SY5Y_Δ_ *_WDR45_* cells than in SH‐SY5Y_WT_ cells (Figure 3f). We also noted a significant reduction in the iron released from ferritin during the 24‐h chase period in the SH‐SY5Y_Δ_ *_WDR45_* cells (Figure 3g, *t*‐test, *t* = 3.573, df = 3, *p* = 0.0375). To further determine whether ferritin‐bound iron leads to cytoplasmic iron deficiency, we have measured levels of the labile iron pool using Calcein‐AM dye, a cell‐permeable fluorescent probe that binds to free iron in the cytoplasm. The turn‐off probe Calcein‐AM dye in the cytosol is quenched upon iron binding. This measurement revealed that SH‐SY5Y_Δ_ *_WDR45_* cells contain lower levels of the labile iron pool than SH‐SY5Y_WT_ cells (Figure 3h, *t*‐test, *t* = 8.283, df = 6, *p* = 0.0002). This response appears to be caused by cytosolic iron deficiency because of the entrapment of iron in ferritin. Taken together, these results suggest that the entrapment of bioactive iron was because of increased ferritin levels, and the impaired ferritin degradation reduced bioavailable iron in SH‐SY5Y_Δ_ *_WDR45_* cells.

### 3.7 Loss of WDR45 leads to lysosomal dysfunction

**FIGURE 4:**
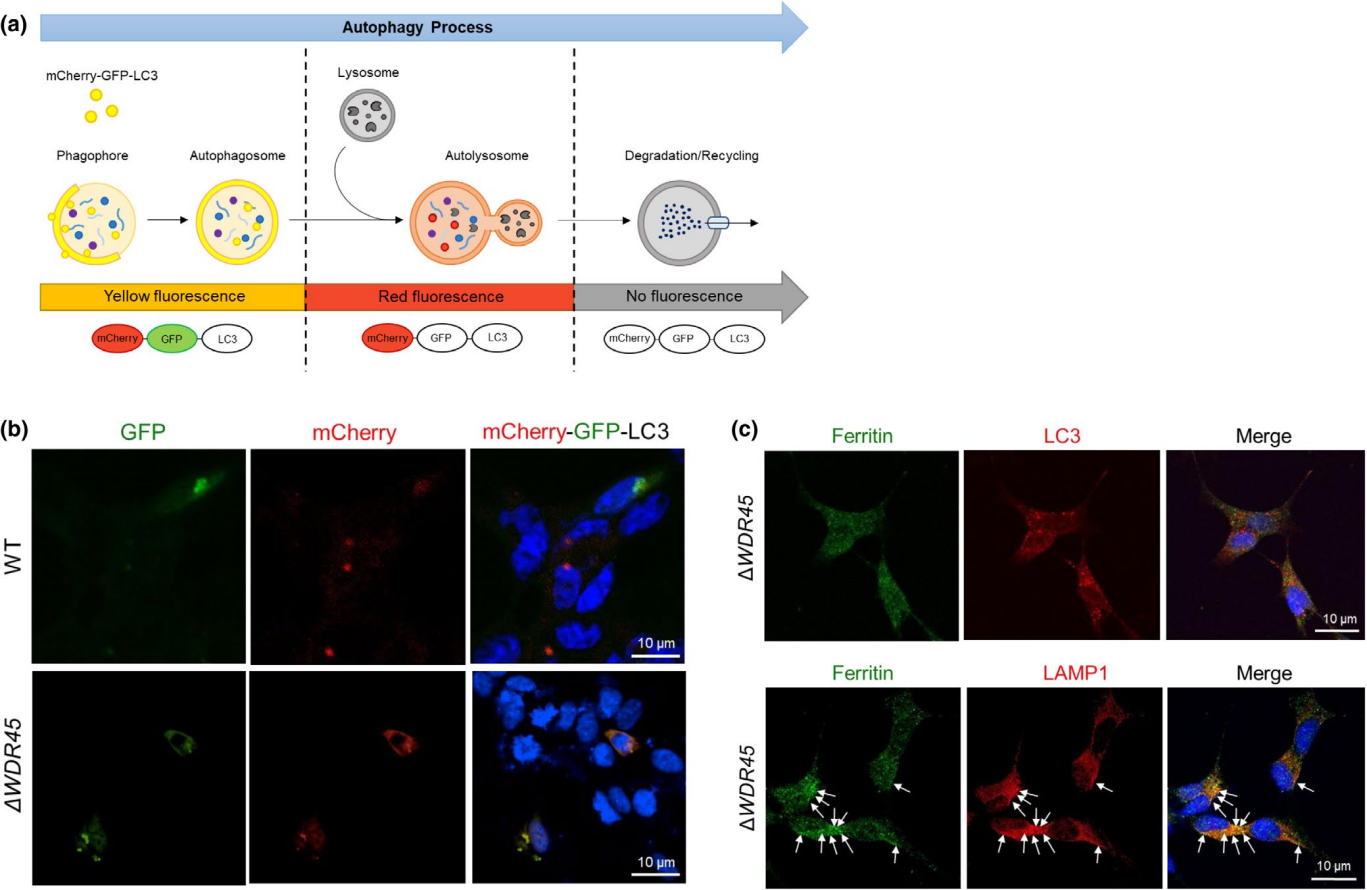
Lysosomal dysfunction in SH‐SY5Y_Δ_ *_WDR45_* cells. (a) Schematic illustration of dual‐fluorescent LC3 assay. The tandem tagged mCherry‐GFP‐LC3 is a pH‐sensitive sensor that is used to monitor the autophagic flux. The GFP tag (green) is acid‐sensitive, whereas the mCherry tag (red) is acid‐insensitive. In autophagosomes, both GFP and mCherry tags emit fluorescent light resulting in yellow fluorescence. By contrast, the fusion of autophagosomes to lysosomes results in acidic autolysosomes where GFP is quenched, and mCherry continues to emit red fluorescence. (b) Representative confocal images of SH‐SY5Y_WT_ or SH‐SY5Y_Δ_ *_WDR45_* cells expressing mCherry‐GFP‐LC3 are shown. SH‐SY5Y_WT_ cells show mostly red fluorescence in lysosomes because of quenching of GFP at low pH, whereas SH‐SY5Y_Δ_ *_WDR45_* cells display a yellow fluorescence in vesicular structures representing autophagosomes. The blue color indicates nuclei counterstained with DAPI. Scale bar: 10 µm. (c) Representative confocal images of ferritin and LC3 or LAMP1 in SH‐SY5Y_Δ_ *_WDR45_* cells. To detect ferritin, the cells were incubated with an anti‐mouse ferritin antibody followed by Alexa Fluor 488‐conjugated secondary antibody (green), indicated by arrows. To detect LC3 or LAMP1, the cells were incubated with anti‐LC3 antibody or anti‐LAMP1 antibody followed by Alexa Fluor 568‐conjugated secondary antibody (red). Blue colors indicate nuclei counterstained with DAPI. Scale bar: 10 µm

To further identify which step of ferritinophagy is defective in SH‐SY5Y_Δ_ *_WDR45_* cells, we monitored the autophagic flux using a chimeric LC3 fused to both GFP (acid sensitive) and mCherry (acid stable) (Kimura et al., 2007; Pankiv et al., 2007). In autophagosomes, the physiological pH allows both GFP (green) and mCherry (red) fluorescence. However, upon fusion with a lysosome, forming an autolysosome, the low pH quenches the GFP signal, permitting only mCherry to fluoresce (Figure 4a). The GFP‐mCherry‐LC3 was expressed in SH‐SY5Y_WT_ and SH‐SY5Y_Δ_ *_WDR45_* cells, and the cells were then analyzed using confocal microscopy. As expected, SH‐SY5Y_WT_ cells exhibited most of the vesicles as red, indicating normal autolysosome formation (Figure 4b). By contrast, SH‐SY5Y_Δ_ *_WDR45_* cells showed predominantly yellow vesicles (Figure 4b), indicating either impaired fusion between autophagosomes and lysosomes, or lysosomal dysfunction including elevated pH. To distinguish between these possibilities, we assessed whether ferritin is accumulated in either autophagosomes or lysosomes in SH‐SY5Y_Δ_ *_WDR45_* cells. We found that SH‐SY5Y_Δ_ *_WDR45_* cells exhibited a substantial overlap of ferritin with the LAMP1 marker corresponding to endosomes and lysosomes but not with LC3, the autophagosome marker (Figure 4c). These data suggest that loss of WDR45 specifically interferes with lysosomal function to degrade ferritin.

### 3.8 Loss of WDR45 increases mitochondrial iron levels and alters the expression of genes involved in oxidative phosphorylation

**FIGURE 5:**
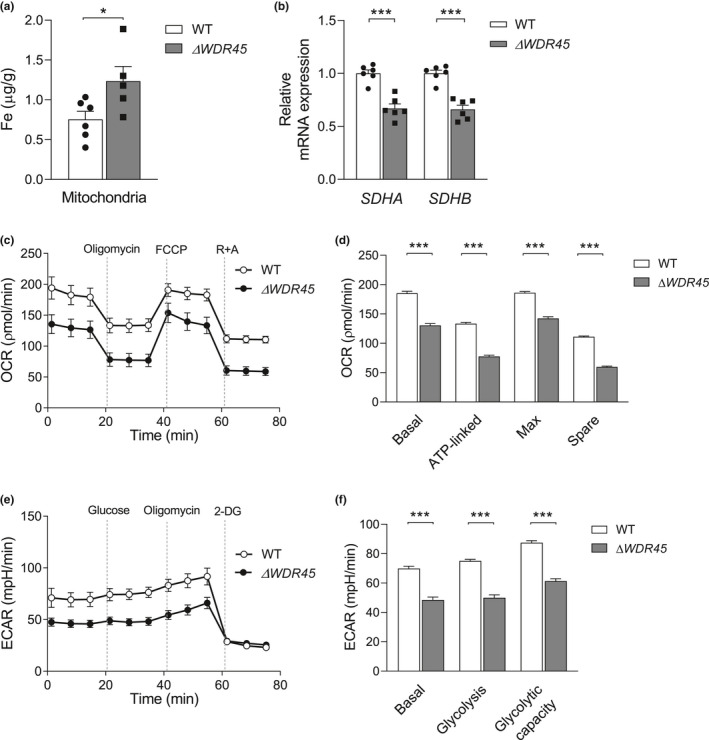
WDR45 deficiency increases mitochondrial iron levels and impairs mitochondrial respiration and glycolytic function. (a) Mitochondrial iron levels were measured from SH‐SY5Y_WT_ and SH‐SY5Y_Δ_ *_WDR45_* cells via ICP‐MS. *n* = 5–6 independent cell culture preparations. (b) Transcription levels of nuclear DNA‐encoded oxidative phosphorylation genes in SH‐SY5Y_WT_ or SH‐SY5Y_Δ_ *_WDR45_* cells. *n* = 6 independent cell culture preparations. (c) Oxygen consumption rate (OCR), an indication of mitochondrial respiration, and (d) basal respiration, ATP turnover, maximal respiration, and spare mitochondrial capacity were measured using a Seahorse Bioscience XF Analyzer. (e) Extracellular acidification rate (ECAR), an indication of glycolysis, and (f) basal glycolysis, glycolysis, and glycolytic capacity were measured using a Seahorse Bioscience XF Analyzer. *n* = 23 independent cell culture preparations. Student's *t*‐test (*p < 0.05 and ***p < 0.001)

When iron is inside cells, it is either stored in ferritin or transferred to organelles such as mitochondria. Mitochondria are responsible for heme synthesis and iron‐sulfur cluster (Fe‐S) biogenesis, which are mainly present in the electron transport chain (Napier et al., 2005; Ponka, 1997). Thus, we hypothesized that WDR45 deficiency might alter iron levels in the mitochondria. Mitochondria were isolated from SH‐SY5Y_Δ_ *_WDR45_* cells, and mitochondrial iron levels were analyzed by ICP‐MS. The iron levels in the mitochondria were 1.6‐fold higher (*t*‐test, *t* = 2.381, df = 9, *p* = 0.0412) in SH‐SY5Y_Δ_ *_WDR45_* cells compared with SH‐SY5Y_WT_ cells (Figure 5a). These data suggest that mitochondrial iron levels, in addition to ferritinophagy, are targets of iron accumulation by WDR45 deficiency.

Mitochondrial abnormalities have been reported in a variety of NBIA subtypes (Arber et al., 2016). The increased level of mitochondrial iron led us to test whether the loss of WDR45 would affect mitochondrial functions. We first examined the mRNA levels in electron transport genes from both mitochondrial DNA (mtDNA) and nuclear DNA (nDNA). The mtDNA genes *MT*‐*ND1* to *MT*‐*ND6*, *MT*‐*CO1*/*COXI* to *MT*‐*CO3*/*COXIII*, *MT*‐*RNR1*/*12S* RNA, and *MT*‐*RNR2*/*16S* RNA are encoded by either the heavy strand or the light strand of mtDNA and play important roles in oxidative phosphorylation. No differences were observed in the expression levels of these mtDNA‐encoded genes in SH‐SY5Y_WT_ or SH‐SY5Y_Δ_ *_WDR45_* cells (Figure S3a). Interestingly, the mRNA expression of the nDNA‐encoded succinate dehydrogenase subunits, *SDHA* and *SDHB*, was markedly reduced in SH‐SY5Y_Δ_ *_WDR45_* cells compared with SH‐SY5Y_WT_ cells (SDHA; *t*‐test, *t* = 6.183, df = 10, *p* = 0.0001, SDHB; *t*‐test, *t* = 6.844, df = 10, *p* < 0.0001) (Figure 5b). We also tested the effects of WDR45 deficiency on mtDNA copy number and mitochondrial mass using MitoTracker Green. No differences were observed in either parameter between SH‐SY5Y_WT_ and SH‐SY5Y_Δ_ *_WDR45_* cells (Figure S3b,c). These results suggest that WDR45 deficiency diminishes the expression of the two nDNA‐encoded genes for mitochondrial subunits involved in oxidative phosphorylation without affecting mtDNA‐encoded subunits.

### 3.9 WDR45 deficiency impairs mitochondrial respiration

The accumulation of mitochondrial iron accompanied by reduced levels of oxidative phosphorylation machinery prompted us to test whether WDR45 deficiency influences the major function of mitochondria. Using Seahorse XF extracellular flux assays, we measured mitochondrial respiratory chain function and oxygen consumption rate (OCR). The results showed that mitochondrial respiratory chain function was significantly decreased in basal respiration, ATP‐linked respiration, and maximal respiration in SH‐SY5Y_Δ_ *_WDR45_* cells compared with SH‐SY5Y_WT_ cells (Figure 5c,d, Basal; *t*‐test, *t* = 12.20, df = 44, *p* < 0.001, ATP‐linked; *t*‐test, *t* = 17.54, df = 44, *p* < 0.001, Max; *t*‐test, *t* = 12.04, df = 44, *p* < 0.001, Spare; *t*‐test, *t* = 27.15, df = 44, *p* < 0.001). Impaired mitochondria function could potentially lead to the metabolic shift to glycolysis (Lee & Yoon, 2015). However, in the present study, SH‐SY5Y_Δ_ *_WDR45_* cells displayed significant reductions in glucose‐induced glycolysis and glycolytic capacity based on measurements of extracellular acidification rates (ECAR) (Figure 5e,f, Basal; *t*‐test, *t* = 8.343, df = 44, *p* < 0.001, Glycolysis; *t*‐test, *t* = 10.98, df = 44, *p* < 0.001, Glycolytic capacity; *t*‐test, *t* = 11.87, df = 44, *p* < 0.001). These results suggest that WDR45 deficiency promotes a general decline in cellular metabolism rather than a shift to glycolysis to compensate for the impaired mitochondrial function.

### 3.10 Loss of WDR45 enhances oxidative damage and induces cell death

**FIGURE 6:**
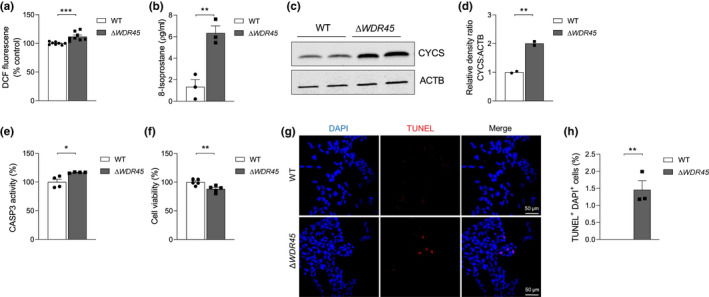
WDR45 deficiency induces ROS production and cell death. (a) Intracellular ROS formation was determined via DCF Fluorescence. *n* = 8 independent cell culture preparations. (b) Oxidative damage was determined by 8‐isoprostane levels. *n* = 3 independent cell culture preparations. (c) Representative immunoblot analysis of CYCS protein levels in total cell lysates from SH‐SY5Y_WT_ and SH‐SY5Y_Δ_ *_WDR45_* cells. Equal loading was verified by immunoblotting with ACTB/actin antibody. (d) Quantification of CYCS protein levels relative to ACTB loading control. *n* = 2 independent cell culture preparations. (e) Percentage of CASP3 activity. *n* = 4 independent cell culture preparations. (f) Cell viability (%) measured using cell counting kit‐8. *n* = 5 independent cell culture preparations. (g) Representative confocal images of TUNEL‐labeled (red) apoptotic cells counterstained with DAPI (blue) in SH‐SY5Y_WT_ or SH‐SY5Y_Δ_ *_WDR45_* cells. The colocalization of TUNEL‐positive and DAPI‐positive cells indicates an apoptotic phenotype in SH‐SY5Y_Δ_ *_WDR45_* cells. Scale bar: 50 µm. (h) Statistical analysis of TUNEL‐positive and DAPI‐positive cell numbers. Three slides per group were used for quantification. *n* = 3 independent cell culture preparations. Student's *t*‐test (*p < 0.05, **p < 0.01, and ***p < 0.001)

The excess of iron promotes the generation of highly reactive free radicals through Fenton chemistry, resulting in oxidative stress and directly damaging DNA or oxidizing lipids and proteins (Uttara et al., 2009). To test whether WDR45 deficiency led to increased oxidative stress, we measured the levels of 2′,7′‐dichlorofluorescein (DCF) as a proxy of oxidative stress (LeBel et al., 1992). The DCF fluorescence was slightly but significantly increased ~1.2 fold (*t*‐test, *t* = 4.185, df = 14, *p* = 0.0009) in SH‐SY5Y_Δ_ *_WDR45_* cells compared with SH‐SY5Y_WT_ cells (Figure 6a). We next measured the oxidative damage levels using an 8‐isoprostane biomarker. The 8‐isoprostane biomarker is a prostaglandin (PG)‐F2‐like compound produced by the free radical‐catalyzed peroxidation of arachidonic acid (van't Erve et al., 2015). The levels of 8‐isoprostane were significantly higher by ~3.7 fold (*t*‐test, *t* = 5.352, df = 4, *p* = 0.0059) in SH‐SY5Y_Δ_ *_WDR45_* cells than in SH‐SY5Y_WT_ cells (Figure 6b).

Iron‐induced oxidative damage can contribute to neurodegeneration (Carocci et al., 2018; Cicero et al., 2017), and cell death is involved with CYCS/cytochrome *c* release from the mitochondria followed by CASP3 (caspase 3) activation (Porter & Janicke, 1999). We determined whether the loss of WDR45 induced CYCS release from mitochondria by analyzing its levels in the cytoplasm. The level of cytoplasmic CYCS was markedly higher in SH‐SY5Y_Δ_ *_WDR45_* cells than in SH‐SY5Y_WT_ cells (Figure 6c,d, *t*‐test, *t* = 13.51, df = 2, *p* = 0.0054). We also detected slightly elevated CASP3 activity (Figure 6e, *t*‐test, *t* = 3.291, df = 6, *p* = 0.0166) and correspondingly reduced cell viability (Figure 6f, *t*‐test, *t* = 3.479, df = 8, *p* = 0.0083) in SH‐SY5Y_Δ_ *_WDR45_* cells compared with SH‐SY5Y_WT_ cells. Lastly, we used terminal deoxynucleotidyl transferase‐mediated dUTP nick end‐labeling (TUNEL) staining to detect apoptotic DNA fragmentation. TUNEL staining showed markedly elevated TUNEL‐positive cells in SH‐SY5Y_Δ_ *_WDR45_* cells relative to SH‐SY5Y_WT_ cells, indicating increased cell death (Figure 6g,h, *t*‐test, *t* = 5.461, df = 4, *p* = 0.0055). Thus, the loss of WDR45 induced oxidative stress and cell death, which may underlie the neurodegeneration observed with WDR45 deficiency (Wan et al., 2019).

### 3.11 Knockdown of ferritin and treatment with a SLC11A2/DMT1 inhibitor reduce iron overload in SH‐SY5Y_Δ_ *_WDR45_* cells

**FIGURE 7:**
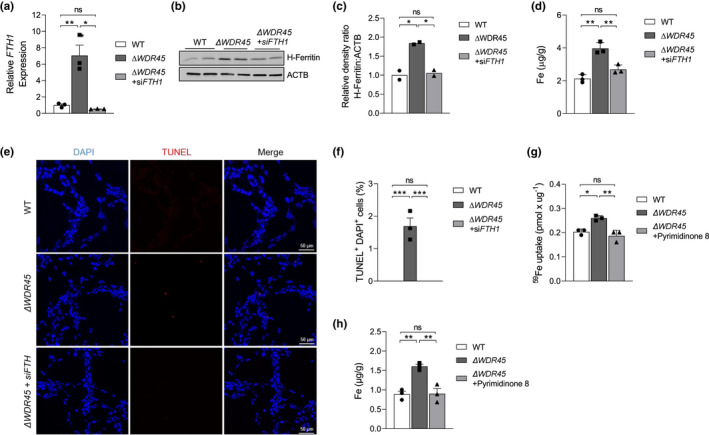
Knockdown of ferritin reduces iron levels in SH‐SY5Y_Δ_ *_WDR45_* cells. (a) SH‐SY5Y_Δ_ *_WDR45_* cells were transfected with control siRNA or anti‐*FTH1* siRNAs. Downregulation of *FTH1* was confirmed by qPCR 48 h after the transfection of siRNA. *n* = 3 independent cell culture preparations. One‐way ANOVA followed by post hoc Tukey's multiple comparisons test (*p < 0.05 and **p < 0.01). (b) Representative immunoblot of H‐ferritin level in total cell lysates isolated from SH‐SY5Y_WT_ and SH‐SY5Y_Δ_ *_WDR45_* cells transfected with control siRNA or anti‐*FTH1* siRNAs. Equal loading was confirmed using ACTB/actin antibody. (c) Quantification of H‐ferritin normalized to ACTB. *n* = 2 independent cell culture preparations. One‐way ANOVA followed by post hoc Tukey's multiple comparisons test (*p < 0.05). (d) Total iron levels were measured by ICP‐MS. *n* = 3 independent cell culture preparations. One‐way ANOVA followed by post hoc Tukey's multiple comparisons test (**p < 0.01). (e) Representative confocal images of TUNEL‐labeled (red) apoptotic cells counterstained with DAPI (blue) in SH‐SY5Y_WT_, SH‐SY5Y_Δ_ *_WDR45_*, or SH‐SY5Y_Δ_ *_WDR45_* cells transfected with si*FTH1* cells. The colocalization of TUNEL‐positive and DAPI‐positive cells indicates an apoptotic phenotype in SH‐SY5Y_Δ_ *_WDR45_* cells. Scale bar: 50 µm. (f) Statistical analysis of TUNEL‐positive and DAPI‐positive cell numbers. *n* = 3 independent cell culture preparations. Three slides per group were used for quantification. One‐way ANOVA followed by post hoc Tukey's multiple comparisons test (**p < 0.01). (g) Cells were treated with 10 µM inhibitor for 10 min prior to the start of and during the ^59^Fe‐NTBI uptake assay for 1 h in pH 6.0 uptake buffer at 37°C. Whole cell ^59^Fe content was determined using a γ‐counter. Each sample was normalized for protein concentration. *n* = 3 independent cell culture preparations. One‐way ANOVA followed by post hoc Tukey's multiple comparisons test (**p < 0.01). (h) Cells were treated with 10 µM inhibitor for 16 h, and total iron levels were measured by ICP‐MS. *n* = 3 independent cell culture preparations. One‐way ANOVA followed by post hoc Tukey's multiple comparisons test (**p < 0.01)

Having established the role of WDR45 in ferritinophagy (Figures 3 and 4), we sought to determine the roles of ferritin in iron accumulation observed in SH‐SY5Y_Δ_ *_WDR45_* cells. To this end, we knocked down *FTH1* (ferritin heavy chain 1) in SH‐SY5Y_Δ_ *_WDR45_* cells using siRNA and measured iron levels. RT‐qPCR analysis showed that *FTH1* was substantially increased by ~7‐fold (*p* < 0.01) in SH‐SY5Y_Δ_ *_WDR45_* cells compared with SH‐SY5Y_WT_ cells, while *FTH1* was effectively reduced to the baseline by the siRNA (Figure 7a, One‐way ANOVA, *F*(2, 6) = 24.01, *p* = 0.0014). Immunoblot analysis confirmed reduction in H‐ferritin expression because of siRNA transfection by ~80% (Figure 7b,c, One‐way ANOVA, *F*(2, 3) = 31.66, *p* = 0.0096). ICP‐MS analysis indicated that SH‐SY5Y_Δ_ *_WDR45_* cells increased iron levels by ~86% (*p* < 0.01) compared with SH‐SY5Y_WT_ cells, whereas the iron concentration returned to normal level upon *FTH1* knockdown (Figure 7d, One‐way ANOVA, *F*(2, 6) = 29.19, *p* = 0.0008). Furthermore, TUNEL staining revealed that knockdown of *FTH1* markedly reduced the number of TUNEL‐positive cells in SH‐SY5Y_Δ_ *_WDR45_* cells, indicating reversed cell death (Figure 7e,f, One‐way ANOVA, *F*(2, 6) = 43.65, *p* = 0.0003). In addition, to confirm a role for SLC11A2/DMT1 in iron accumulation observed in SH‐SY5Y_Δ_ *_WDR45_* cells, pyrimidinone 8 was used as a pharmacological inhibitor of this transporter (Montalbetti et al., 2015). Incubation with pyrimidinone 8 for 10 min prior to the start of and during the ^59^Fe‐NTBI uptake assay reduced ^59^Fe‐NTBI uptake by SH‐SY5Y_Δ_ *_WDR45_* cells (Figure 7g, One‐way ANOVA, *F*(2, 6) = 17.35, *p* = 0.0032). We also confirmed that pyrimidinone 8 treatment reduced the iron concentration to a normal level in SH‐SY5Y_Δ_ *_WDR45_* cells (Figure 7h, One‐way ANOVA, *F*(2, 6) = 19.13, *p* = 0.0025). These combined data indicate that elevated ferritin level and SLC11A2/DMT1‐mediated NTBI uptake pathway contribute to iron accumulation in SH‐SY5Y_Δ_ *_WDR45_* cells.

## 4 DISCUSSION

**FIGURE 8:**
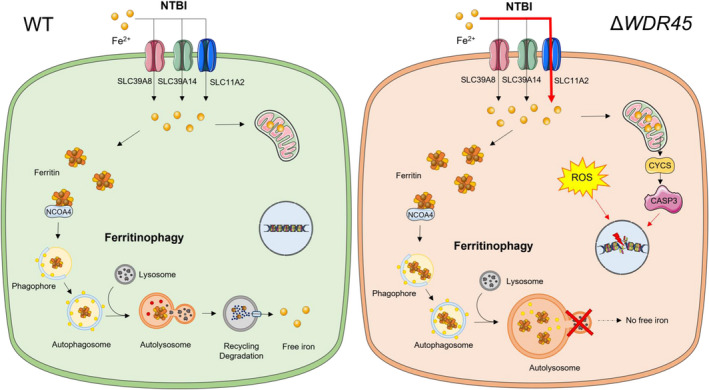
Schematic model for the impact of WDR45 deficiency on iron accumulation, ferritinophagy, and cell death. WDR45‐deficient cells accumulate iron via the nontransferrin‐bound iron pathway (NTBI). The increased total iron levels were accompanied by an increase in the iron storage protein ferritin. WDR45 deficiency impairs ferritinophagy, the autophagic degradation of ferritin. Elevated iron levels were also detected in the mitochondria, which may impair mitochondrial function, elevate reactive oxygen species, and increase cell death

The goal of this study was to identify the major proteins and pathways involved in iron accumulation as a result of WDR45 deficiency and how the altered iron uptake and metabolism contribute to neurodegeneration. We provided several lines of evidence for the major pathways leading to brain iron overload in WDR45 deficiency, and our results shed light on the mechanisms by which WDR45 deficiency affects iron‐induced neurodegeneration (Figure 8). First, our ^59^Fe flux study revealed that WDR45 deficiency induced significant changes in cellular iron uptake by acquiring NTBI in the more acidic environment rather than TBI. Second, we found that WDR45 deficiency led to the upregulation of the NTBI uptake transporter SLC11A2/DMT1−IRE and the downregulation of iron exporter SLC40A1/FPN levels, which may underlie neuronal iron accumulation. Third, we showed that cellular iron overload in WDR45 deficiency was associated with impaired ferritinophagy. Fourth, WDR45 deficiency leads to mitochondrial iron accumulation, altered mitochondrial metabolism, and enhanced ROS production, thus potentially contributing to neuronal cell death and neurodegeneration.

The effects of WDR45 deficiency on iron transport have not been directly examined. We found that the SH‐SY5Y_WT_ cells in our study took up iron presented in both the TBI and NTBI forms. Although TBI is a constituent of brain interstitial fluid, our ^59^Fe‐NTBI uptake results revealed that the levels of NTBI uptake were much higher in SH‐SY5Y_Δ_ *_WDR45_* cells compared with SH‐SY5Y_WT_ cells, suggesting that NTBI acts as a major substrate for iron uptake by SH‐SY5Y_Δ_ *_WDR45_* cells. Correspondingly, the entry of NTBI into the cells was associated with increased levels of ferritin expression. When iron is inside cells, it is either deposited into ferritin or incorporated into the mitochondria; otherwise, iron should be subsequently exported by SLC40A1/FPN, the only known cellular iron exporter in mammals (Abboud & Haile, 2000; McKie et al., 2000). We found that SH‐SY5Y_Δ_ *_WDR45_* cells displayed significantly reduced SLC40A1/FPN expression along with increased HAMP/hepcidin expression, suggesting that the reduced iron export function in response to elevated expression of HAMP is also responsible for cellular iron sequestration as a result of WDR45 deficiency. In contrast to the increased levels of ferritin and HAMP that we observed, a study on patient‐derived *WDR45* mutant fibroblast cells reported reduced levels of L‐ferritin and HAMP following elevated intracellular iron levels (Seibler, 2018). These inconsistent observations may be explained by the different cell types used in the studies (fibroblast vs. neuroblastoma cells) or the nature of *WDR45* mutations (hypomorphic mutation in the patient cells vs. the null mutations in our study).

One striking result of our study was that in addition to iron, the zinc and copper levels in SH‐SY5Y_Δ_ *_WDR45_* cells under basal conditions appeared to be affected. This result was noteworthy because previous studies on humans, mice, or cell lines with WDR45 deficiency have not directly determined concentrations of non‐iron metals such as zinc and copper. In particular, because iron and zinc have similar atomic radiuses and oxidation states, they were postulated to share common transport machineries, such as SLC11A2/DMT1 and SLC40A1/FPN. Studies on *Xenopus* oocytes have shown that SLC11A2/DMT1 not only mediates uptake of ferrous iron (Fe^2+^) but also interacts with other divalent metals, including Cd^2+^, Co^2+^, Mn^2+^, Ni^2+^, VO^2+^, and Zn^2+^ (Illing et al., 2012; Shawki et al., 2012). SLC40A1/FPN expression in *Xenopus* oocytes also stimulated the efflux of Fe^2+^, Zn^2+^, and Co^2+^ (Mitchell et al., 2014). These studies suggest that the upregulation of SLC11A2/DMT1 and downregulation of SLC40A1/FPN by WDR45 deficiency may contribute to the accumulation of zinc in addition to iron. Although SLC39A8/ZIP8 and SLC39A14/ZIP14 are capable of transporting zinc and iron (Ji & Kosman, 2015; Liuzzi et al., 2006; Wang et al., 2012), the downregulation of these proteins in SH‐SY5Y_Δ_ *_WDR45_* cells makes it unlikely to contribute to cellular zinc accumulation. Although the simplest hypothesis is that SLC11A2/DMT1 or SLC40A1/FPN mediate the uptake or export of zinc in WDR45‐deficient cells, WDR45 loss and iron overload may influence other zinc homeostatic mechanisms. Further studies are necessary to define the role of WDR45 in the transport of zinc or copper and whether and how they contribute to neurological issues in human patients.

Previous studies have reported autophagic dysfunction in *WDR45* mutant patient‐derived lymphoblast cells (Saitsu et al., 2013) and *wdr45*‐KO mice (Wan et al., 2019; Zhao et al., 2015). However, lymphoblast cells from patients with static encephalopathy of childhood with neurodegeneration in adulthood (SENDA), now renamed BPAN, showed a reduction, rather than a block, in autophagic flux (Saitsu et al., 2013). Furthermore, the study by Zhao et al. (2015) did not analyze flux using standard methodologies (Klionsky et al., 2008); the authors compared WT and mutant cells with the lysosomal inhibitor bafilomycin A_1_, but they did not examine either cell line in the same experiment in the absence and presence of inhibitor, which is necessary to determine flux. Finally, the study by Wan et al. (2019) failed to examine autophagic flux at all, and simply measured the steady‐state levels of LC3‐II and SQSTM1. Thus, these studies suggest at most a partial block in autophagic flux. We examined the autophagic flux by measuring the levels of LC3‐II and SQSTM1/p62. Unlike previous studies, our data revealed similar levels of LC3‐II and SQSTM1/p62 between SH‐SY5Y_WT_ and SH‐SY5Y_Δ_ *_WDR45_* cells under basal conditions (Figure S2a). Moreover, LC3‐II levels were similar in SH‐SY5Y_WT_ and SH‐SY5Y_Δ_ *_WDR45_* cells even after Torin 1 treatment and the concomitant treatment with CQ. Similarly, we did not detect any substantial differences in SQSTM1/p62 levels between the two groups. These data suggest that WDR45 may not be essential for autophagosome formation. Similar to our results, a recent study using the mouse N2a/Neuro2a neuroblastoma cell line failed to detect a defect in autophagy in *wdr45* KO cells (in contrast to the previous study from some of the same authors (Zhao et al., 2015)), but did observe dysfunctional autophagy in *wdr45 wdr45b* double KO cells (Ji et al., 2021). These data suggest a partial overlap in the function of the two homologous gene products, similar to the result seen with the homologous yeast proteins Atg18 and Atg21 (Nair et al., 2010). Notably, we observed that overall levels of LC3‐I and LC‐II were reduced in SH‐SY5Y_Δ_ *_WDR45_* cells compared with SH‐SY5Y_WT_ cells, raising the possibility that WDR45 may play some role in the biogenesis of autophagosomes. Importantly, we think that SH‐SY5Y_Δ_ *_WDR45_* cells are partially defective in lysosomal function, which may account for the phenotypes observed in all of these studies.

Mitochondria are one of the major cellular storage organelles for iron (Arber et al., 2016). Accordingly, mitochondrial iron levels should be tightly regulated to provide sufficient iron for numerous cellular processes that use iron as a co‐factor, including the electron transport chain, while guarding against the generation of radicals induced by an excess of redox‐active iron (Urrutia et al., 2014). We found that WDR45 deficiency leads to mitochondrial iron accumulation, impaired mitochondrial respiration, elevated ROS, and increased neuronal cell death. Consistent with our findings, mitochondrial iron accumulation has been implicated in many neurodegenerative diseases (Mena et al., 2015). In addition, we found markedly reduced levels of nDNA‐encoded SDH subunits without affecting the levels of mtDNA‐encoded genes in SH‐SY5Y_Δ_ *_WDR45_* cells (Figure 5b). Inhibition of SDH leads to neuronal death in caudate and putamen nuclei and symptoms of Huntington disease (Túnez et al., 2010). Another competitive SDH inhibitor malonate can cause neuronal injuries and degeneration (Beal et al., 1993) through mitochondrial membrane potential collapse and subsequent release of proapoptotic factors such as CYCS/cytochrome *c* (Fernandez‐Gomez et al., 2005). Future studies are warranted to test the mitochondrial iron accumulation and SDH as targets for therapeutic interventions in patients with BPAN.

Our study is the first to link WDR45 to ferritinophagy. The SH‐SY5Y_Δ_ *_WDR45_* cells showed impaired release of ^59^Fe from ferritin and the sequestration of iron‐loaded ferritin in lysosomes (Figure 4). What are the mechanisms by which WDR45 regulates ferritinophagy? NCOA4 is a receptor protein that links ferritin‐containing cargo to phagophores (Mancias et al., 2014). However, we found that WDR45 deficiency did not alter NCOA4 levels. The worm ortholog of WDR45, EPG‐6, is essential for an early step of autophagosome formation (Lu et al., 2011). However, our confocal microscopy data demonstrated that WDR45 deficiency did not affect the formation of autophagosomes but instead inhibited amphisome–lysosome fusion or lysosomal function. Thus, these data suggest that WDR45 plays an essential role in a late step of ferritinophagy involving lysosomal function. Another key question concerns how impaired ferritinophagy is linked to the increased iron uptake by SLC11A2/DMT1 in the WDR45‐deficient cells. We speculate that the accumulation of ferritin‐bound iron induces cytoplasmic iron deficiency (Figure 3h), which would upregulate SLC11A2/DMT1 expression, thereby leading to further iron incorporation into SH‐SY5Y_Δ_ *_WDR45_* cells. In support of this idea, ferritin knockdown and SLC11A2/DMT1 inhibitor alleviated the iron accumulation in WDR45‐deficient cells (Figure 7). However, the present study does not rule out the possibility of alternative mechanisms such as upregulation of ferritin expression in response to oxidative stress. The expression of ferritin is primarily regulated through post‐transcriptional, iron‐dependent machinery based on the interaction by the IRPs and IRE located on the target mRNAs (Leibold & Guo, 1992). Oxidative stress has been shown to increase the synthesis of both subunits of ferritin as well as the activity of IRP in HeLa cells (Orino et al., 2001). Conversely, overexpression of either ferritin subunit reduces ROS formation in response to oxidative stress (Orino et al., 2001). Moreover, ER stress activation was reported to induce the expression of ferritin in HepG2 cells (Oliveira et al., 2009). These studies led us to postulate the alternative hypothesis that excess iron‐induced oxidative stress may be involved with the upregulation of ferritin in WDR45‐deficient cells. Iron is needed for cell survival via many cellular processes such as oxidative metabolism. Meanwhile, iron is also needed for programed cell death mediated by ferroptosis. In our study, impaired ferritinophagy in WDR45 mutant cells was accompanied by increased cell death. These data suggest that reduced labile iron as a result of the impaired ferritinophagy had a larger impact on the pro‐survival role of iron compared with ferroptosis.

## CONFLICT OF INTEREST

The authors declare no competing interest.

## AUTHOR CONTRIBUTIONS

Y.A.S conceived and designed the study. L.A., E.K.C., H.K., and Y.A.S. performed research; L.A., E.K.C., H.K., T.L., S.I., D.J.K., and Y.A.S. analyzed and interpreted data; and L.A. and Y.A.S. wrote the paper. Y.A.S was responsible for funding acquisition.

### OPEN RESEARCH BADGES

This article has received a badge for Open Materials because it provided all relevant information to reproduce the study in the manuscript. More information about the Open Science badges can be found at https://urldefense.com/v3/__https://cos.io/our‐services/open‐science‐badges/__;!!N11eV2iwtfs!5ezBrT68HKDaTcKFB_tgQSbQctmf3Js9zk_hsOVYVIezTUXnccegybbzifUxMQ$

## Supporting information

Supplementary Material

Table S1

## Data Availability

The data that support the findings of this study are available from the corresponding author upon reasonable request.
