# Supplementary material for: A neurodegeneration gene, WDR45, links impaired ferritinophagy to iron accumulation
Source: J Neurochem. 2021 Dec 8;160(3):356–75. doi: 10.1111/jnc.15548 (PMC8811950; doi:10.1111/jnc.15548)
Supplement: Supplementary file 1 — Supplementary Material [file JNC-160-356-s001.pdf]

Supplementary Information

A neurodegeneration gene, *WDR45*, links impaired ferritinophagy to iron accumulation

Luisa Aring[1,†], Eun-Kyung Choi[1,†], Huira Kopera[2,3], Thomas Lanigan[3,4], Shigeki Iwase[2], Daniel J Klionsky[5], and Young Ah Seo [1,‡]

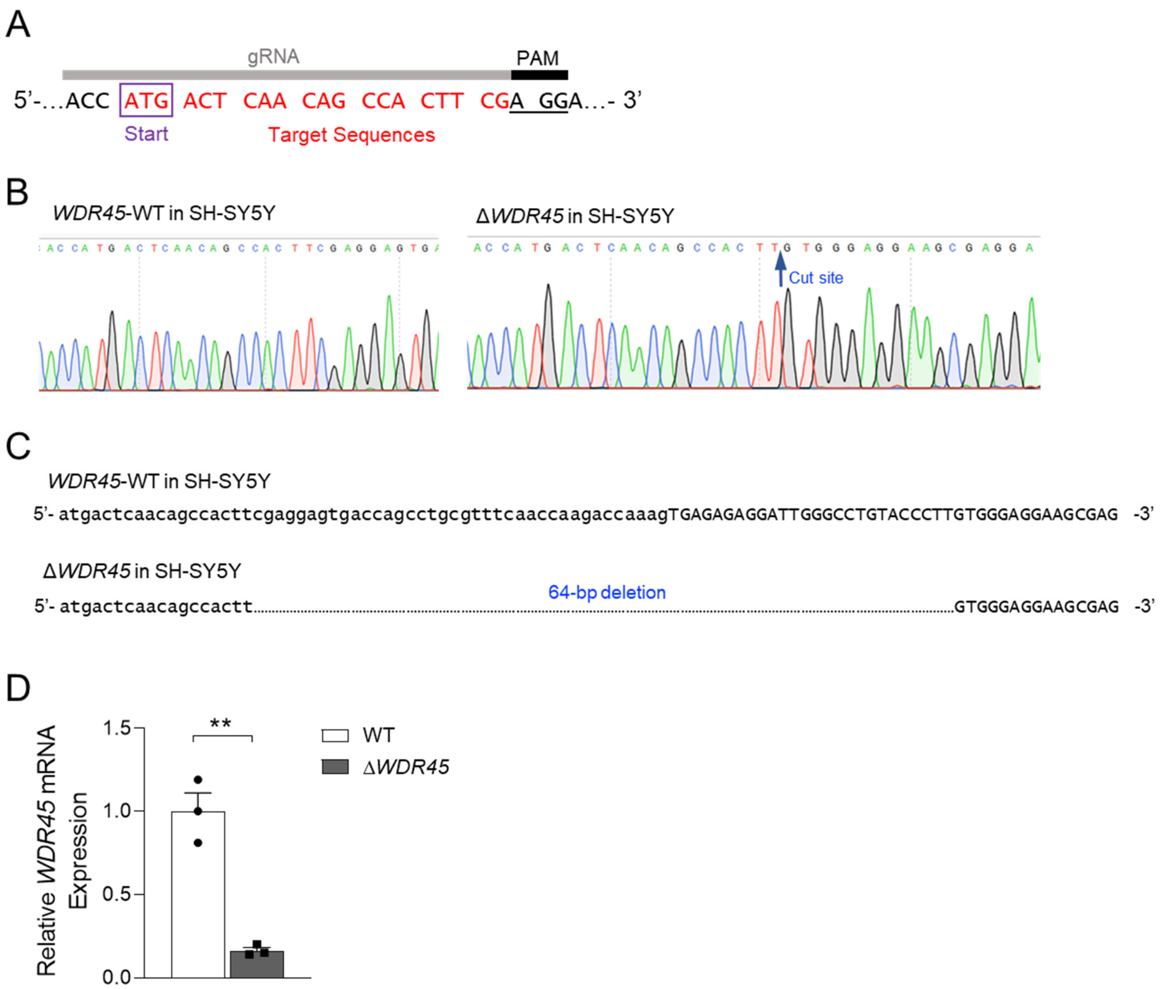

Fig. S1. *WDR45*-knockout in SH-SY5Y cells. (A) The gRNA targeting the WDR45 coding sequence is in red, and the PAM sequence is underlined. (B) The sequencing chromatograms show the genomic DNA sequence of wild-type *WDR45* (left panel) and CRISPR-edited *WDR45* sequence in SH-SY5Y cells (right panel); the CRISPR cut site is noted. Both alleles appear to have the same CRISPR edit. (C) The SH-SY5Y$_{\Delta WDR45}$ cell line has a 64-bp deletion that spans an intron splice donor site. Intronic sequences are capitalized. (D) Expression of *WDR45* in SH-SY5Y$_{\Delta WDR45}$ cells. n = 3 independent cell culture preparations. ** $P < 0.01$.

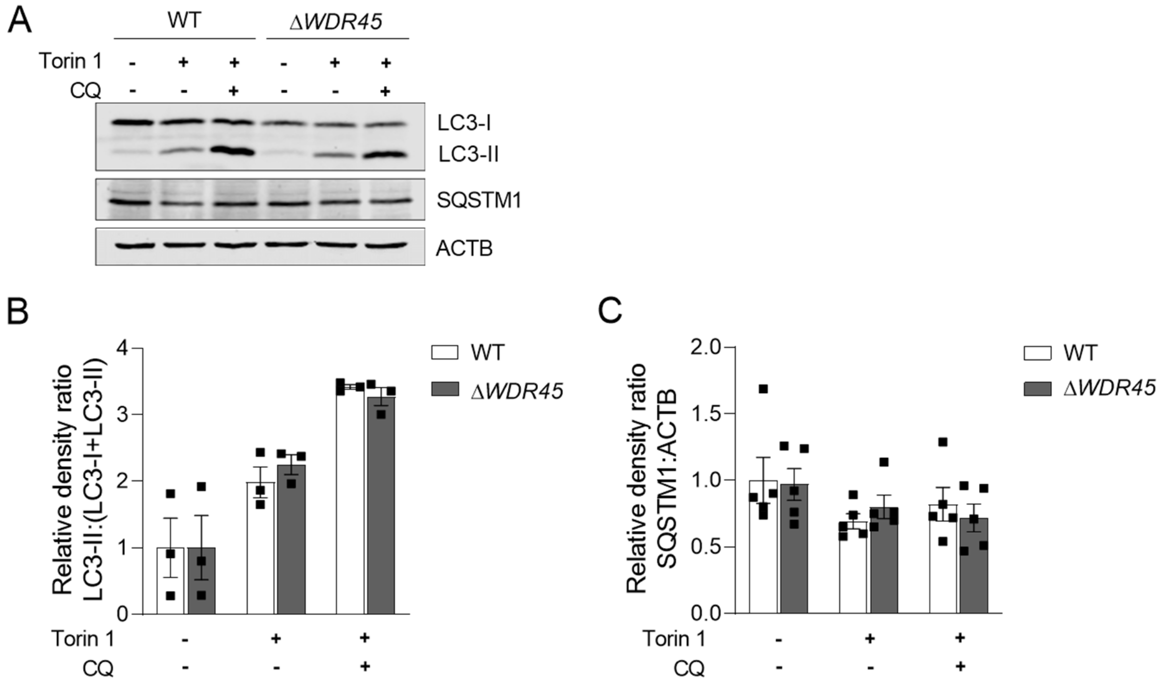

Fig. S2. Autophagic flux in SH-SY5Y$_{\Delta WDR45}$ cells. (A) Representative immunoblot of LC3-I, LC3-II, and SQSTM1/p62 levels in total cell lysates isolated from SH-SY5Y$_{WT}$ and SH-SY5Y$_{\Delta WDR45}$ cells treated without or with 500 nM Torin1 in the presence or absence of 20 µM chloroquine (CQ) for 2 h. Equal loading was verified by immunoblotting with ACTB/actin antibody. (B, C) Quantification of LC3-II and SQSTM1/p62 relative protein after normalization with total LC3 (LC3-1 + LC3-II) and ACTB, respectively. n = 3 independent cell culture preparations.

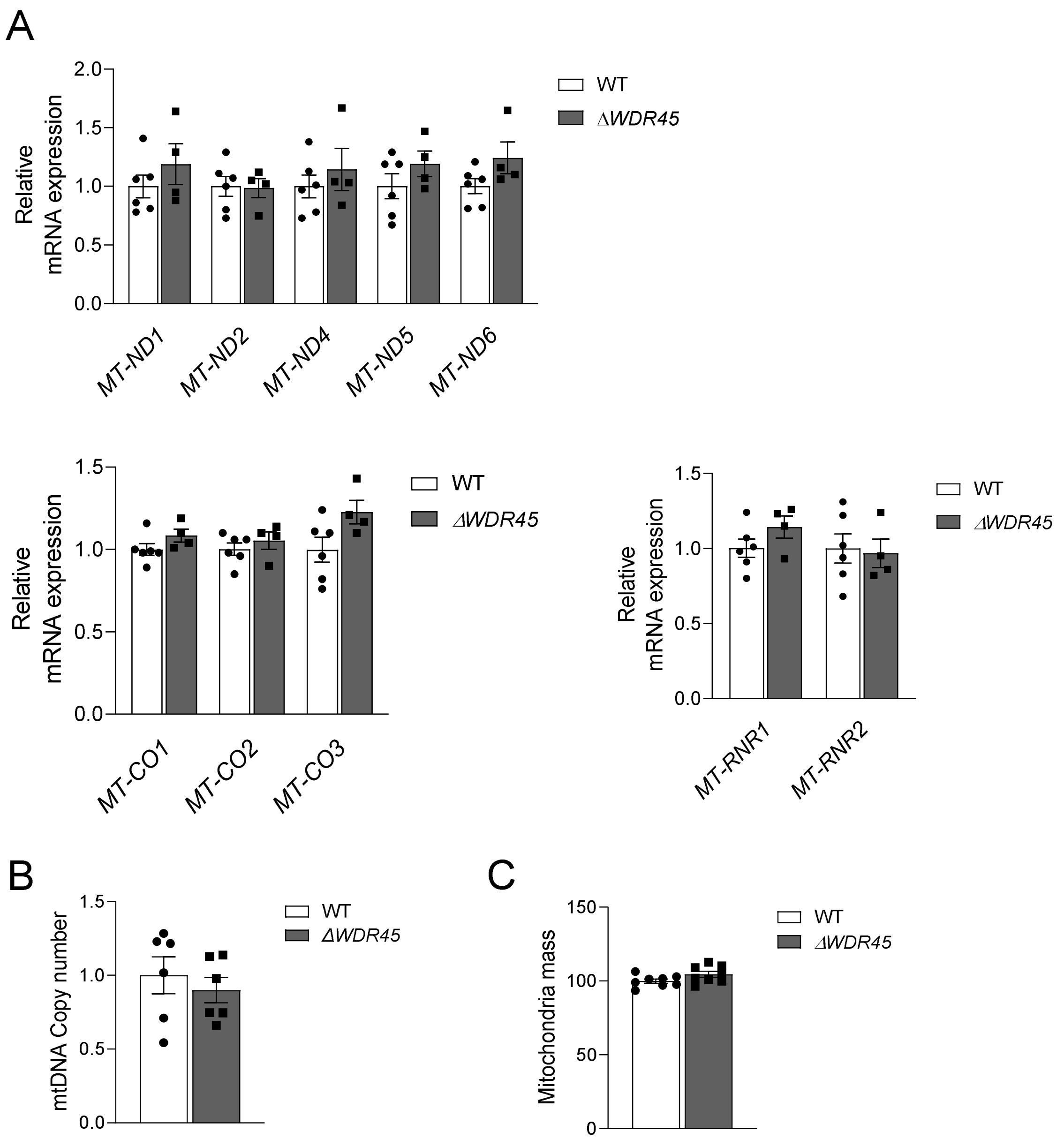

Fig. S3. Gene expression involved in oxidative phosphorylation in SH-SY5Y$_{\Delta WDR45}$ cells. (A) Transcription levels of mitochondrial DNA-encoded oxidative phosphorylation genes in SH-SY5Y$_{WT}$ or SH-SY5Y$_{\Delta WDR45}$ cells. n = 4-6 independent cell culture preparations. (B) mtDNA copy number and (C) mitochondrial mass in SH-SY5Y$_{WT}$ and SH-SY5Y$_{\Delta WDR45}$ cells. n = 6-8 independent cell culture preparations.

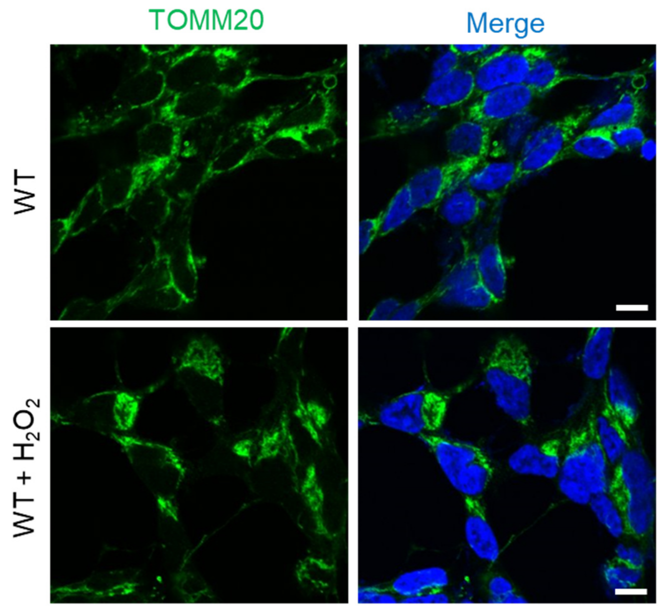

Fig. S4. Representative confocal images of outer mitochondrial membrane marker TOMM20 (green) counterstained with DAPI (blue) in SH-SY5Y$_{WT}$ treated with H_2O_2. Scale bar: 10 µm.

Table S1. Primer sequences used in this study.

Gene	Forward primer	Reverse primer
WDR45	CCAGCCTGGAGAAGCAAC	GCTCCACCAGTTTCTCCTTG
TFRC	CCTGCACGTCGCTTATA	ACCGAGTTTTGAGCGCTGTC
SLC39A8	TCCTGCACCTTGTCTCTCCT	GCCCAACATAGCAGGAACAT
SLC39A14	CATTTGGTTTCAACCCTCTGGAAC	TTTCAGCCAGTAGCAAGCACTCTG
SLC11A2+IRE	GAGCCAGTGTGTTTCTATGG	CCTAAGCCTGATAGAGCTAG
SLC11A2-IRE	GGGAAGGGTGTTCAAAACTG	CAATGCAGCACGGAAAACTG
SLC40A1	TACTTGTGCCTCCCAGATGG	ATGGAACCACTCAGTCCCTG
HAMP	CCACTTCCCCATCTGCATTT	GCAGCACATCCCACACTTTG
sCP	CTCACAATGCACGTGGGAGA	CAGCCAGATTTGGTGTCTTCA
APP	TGAGCGCATGAATCAGTCTC	CCAGGCTGAACTCTCCATTC
FTH1	ACTGATGAAGCTGCAGAACC	GTCACCCAATTCTTTGATGG
MT-ND1	CCTAGGCCTCCTATTTATTC	GAATGATGGCTAGGGTGA
MT-ND2	CTACGCCTAATCTACTCCAC	CTTTGAAGGCTCTTGGTCTG
MT-ND4	GGACTCCACTTATGACTCCC	GGTTGAGAATGAGTGTGAGGC
MT-ND5	CTATCACCACTCTGTTCGCAG	GTGGTTGGTTGATGCCGATTG
MT-ND6	CTAAAACACTCACCAAGACC	GGAATGATGGTTGTCTTTGG
MT-CO1	GATTTTTCGGTCACCCTGAAG	CTCAGACCATACCTATGTATC
MT-CO2	CTATCCTGCCCGCCATCATC	GATTAGTCCGCCGTAGTCGG
MT-CO3	CACATCCGTATTACTCGCATC	GAAGTACTCTGAGGCTTGTAG
MT-RNR1/12SRNA	CACTACGAGCCACAGCTTAA	TCAGGGTTTGCTGAAGATGG
MT-RNR2/16SRNA	GGCATGCTCATAAGGAAAGG	GGCCGTTAAACATGTGTCAC
18S RNA	GAGGTAGTGACGAAAAATAACAAT	TTGCCCTCCAATGGATCCT
SDHA	CCTTTCTGAGGCAGGGTTTA	AGAGCAGCATTGATTCCTCC
SDHB	ACCTTCCGAAGATCATGCAGA	GTGCAAGCTAGAGTGTTGCCT
HBB/β-globin	CCTTTGTTCCCTAAGTCCAA	CCTCACCTTCTTTCATGGAG
