# Supplementary material for: A neurodegeneration gene, WDR45, links impaired ferritinophagy to iron accumulation
Source: J Neurochem. 2021 Dec 8;160(3):356–75. doi: 10.1111/jnc.15548 (PMC8811950; doi:10.1111/jnc.15548)
Supplement: Supplementary file 2 — Table S1 [file JNC-160-356-s002.docx]

# Table S1. Primer sequences used in this study.

| Gene | Forward primer | Reverse primer |
| --- | --- | --- |
| *WDR45*  *TFRC*  *SLC39A8*  *SLC39A14*  *SLC11A2+IRE*  *SLC11A2-IRE*  *SLC40A1*  *HAMP*  *sCP*  *APP*  *FTH1*  *MT-ND1*  *MT-ND2*  *MT-ND4*  *MT-ND5*  *MT-ND6*  *MT-CO1*  *MT-CO2*  *MT-CO3*  *MT-RNR1/12SRNA*  *MT-RNR2/16SRNA*  *18S RNA*  *SDHA*  *SDHB*  *HBB/β-globin* | CCAGCCTGGAGAAGCAAC  CCTGCACGTCGCTTATA  TCCTGCACCTTGTCTCTCCT  CATTTGGTTTCAACCCTCTGGAAC  GAGCCAGTGTGTTTCTATGG  GGGAAGGGTGTTCAAAACTG  TACTTGTGCCTCCCAGATGG  CCACTTCCCCATCTGCATTT  CTCACAATGCACGTGGGAGA  TGAGCGCATGAATCAGTCTC  ACTGATGAAGCTGCAGAACC  CCTAGGCCTCCTATTTATTC  CTACGCCTAATCTACTCCAC  GGACTCCACTTATGACTCCC  CTATCACCACTCTGTTCGCAG  CTAAAACACTCACCAAGACC  GATTTTTCGGTCACCCTGAAG  CTATCCTGCCCGCCATCATC  CACATCCGTATTACTCGCATC  CACTACGAGCCACAGCTTAA  GGCATGCTCATAAGGAAAGG  GAGGTAGTGACGAAAAATAACAAT  CCTTTCTGAGGCAGGGTTTA  ACCTTCCGAAGATCATGCAGA  CCTTTGTTCCCTAAGTCCAA | GCTCCACCAGTTTCTCCTTG  ACCGAGTTTTGAGCGCTGTC  GCCCAACATAGCAGGAACAT  TTTCAGCCAGTAGCAAGCACTCTG  CCTAAGCCTGATAGAGCTAG  CAATGCAGCACGGAAAACTG  ATGGAACCACTCAGTCCCTG  GCAGCACATCCCACACTTTG  CAGCCAGATTTGGTGTCTTCA  CCAGGCTGAACTCTCCATTC  GTCACCCAATTCTTTGATGG  GAATGATGGCTAGGGTGA  CTTTGAAGGCTCTTGGTCTG  GGTTGAGAATGAGTGTGAGGC  GTGGTTGGTTGATGCCGATTG  GGAATGATGGTTGTCTTTGG  CTCAGACCATACCTATGTATC  GATTAGTCCGCCGTAGTCGG  GAAGTACTCTGAGGCTTGTAG  TCAGGGTTTGCTGAAGATGG  GGCCGTTAAACATGTGTCAC  TTGCCCTCCAATGGATCCT  AGAGCAGCATTGATTCCTCC  GTGCAAGCTAGAGTGTTGCCT  CCTCACCTTCTTTCATGGAG |
